# Flash Communication: Bismuthinidenes as Lewis Bases in Reversible C(sp^3^)–O Bond Cleavage

**DOI:** 10.1021/acs.organomet.5c00260

**Published:** 2025-08-20

**Authors:** Davide Spinnato, Hye Won Moon, Markus Leutzsch, Nils Nöthling, Josep Cornella

**Affiliations:** 28314 Max-Planck-Institut für Kohlenforschung, Kaiser-Wilhelm-Platz 1, 45470 Mülheim an der Ruhr, Germany

## Abstract

Herein, we describe a unique example of a low-valent monocoordinated Bi­(I) complex acting as a Lewis base partner in a FLP activation reaction. The interplay between a bulky and rigid Bi­(I) and the Lewis acidic tris­(pentafluorophenyl)­borane (BCF) enables the opening of tetrahydrofuran (THF), delivering a zwitterionic Bi­(+)/B(−) product, which could be isolated and characterized spectroscopically and through SC-XRD. The zwitterionic bismuth complex exhibits remarkable spectroscopic features in the visible range, namely an LMCT band corresponding to the transition from the aromatic fluorene to the highly electron-poor cationic bismuth center. Interestingly, excitation of this band results in ring-closing C–O bond formation and subsequent recovery of the Bi­(I), thus constituting a photoreversible FLP activation with bismuth.

## Graphical abstract

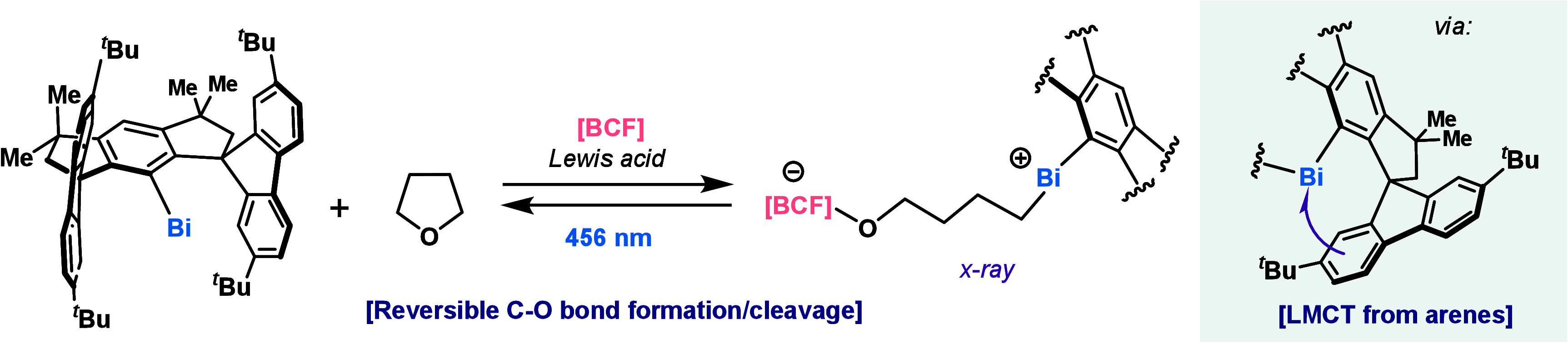

Frustrated Lewis pairs (FLPs) comprising of a sterically encumbered Lewis base (LB) and Lewis acid (LA) represent a class of versatile catalysts for activating small molecules under mild, transition-metal-free conditions.^1^ The concept, pioneered by Stephan and Erker, leverages the inability of the LB and LA to form a stable dative bond due to steric and electronic factors.^2^ Steric frustration arises when bulky substituents prevent the spatial alignment of frontier molecular orbitals (FMOs), while electronic frustration results from an energetic mismatch between the FMOs of the LA and LB, weakening their interaction.^3^ This dual frustration enables FLPs to activate molecules such as H_2_, CO_2_, and alkenes, with applications spanning from small molecule activation to organic synthesis.^4^ Traditionally, FLPs capitalize on the basicity of the lone pair of trisubstituted nitrogen or phosphorus compounds.^1,2^ However, examples with heavier pnictogens remain scarce, despite the potential benefit of a larger energetic mismatch between the FMOs when using lighter Lewis acid centers such as trivalent boron (B). Recent advances in low-valent main-group chemistry, particularly with bismuth (Bi), have opened new avenues in catalysis.^5^ Indeed, low-valent Bi­(I) complexes bear a lone pair in the 6p orbital, which have been extensively exploited in catalytic redox processes or Lewis-base catalysis (e.g., compound **1**, Figure 1).^5^ Yet, bismuthinidenes have been underexplored in FLP chemistry. Unlike conventional nitrogen- or phosphorus-based LBs, bismuth­(I) complexes offer a combination of σ-donation and π-acceptor capabilities, potentially enabling novel reactivity paradigms.

**Figure 1:**
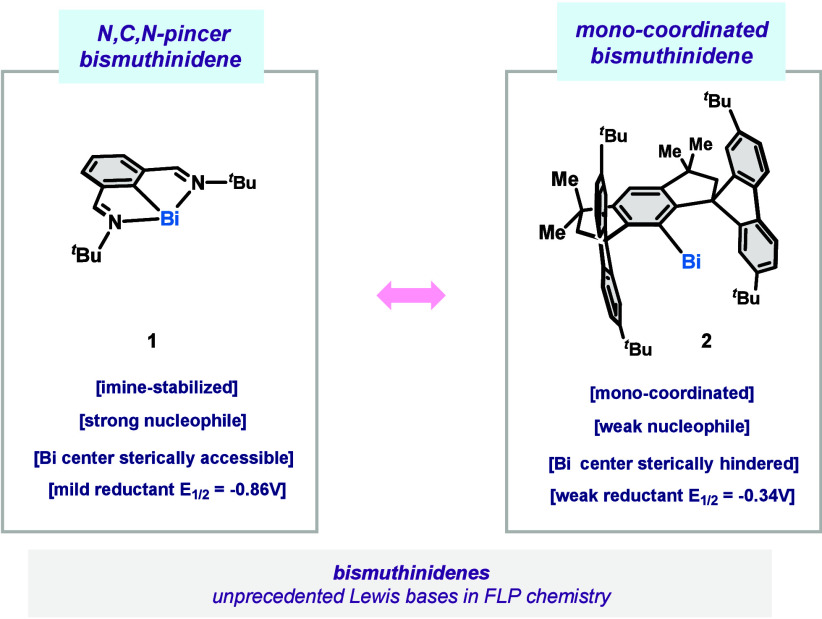
Comparison between selected bismuthinidenes.

In this work, we report an example of a reactive frustrated Lewis pair comprising a low-valent Bi­(I) complex as the Lewis base. The pairing of a monocoordinated bismuthinidene (compound **2**, Figure 1)^6^ with a suitable Lewis acid for molecular activation highlights the potential of such compounds as Lewis bases in the area of FLP catalysis.

We selected the ring-opening of THF as the model reaction to benchmark reactivity. Indeed, when THF is coordinated with BCF, the C­(sp^3^)–O bond can be cleaved by various pnictogenic Lewis bases, including phosphines or amines (Figure 2a).^7^ With these precedents in hand, we decided to test two Bi­(I) complexes in a similar context. When the venerable Dostál Bi­(I) (**1**)^8^ complex was mixed with tris­(pentafluorophenyl)­borane (BCF) in the presence of 5.0 equiv of THF in toluene at 35 °C, an intractable glue was rapidly formed (Figure 2b). The latter presumably arises from uncontrolled polymerization of THF, accompanied by Bi degradation. However, when tricoordinated **1** was replaced by the bulky and rigid monocoordinated **2** under the same reaction conditions, clean formation of **3** was observed by ^1^H NMR (Figure 2c). Compound **3** could be isolated in 75% as a red solid and its structure was unambiguously determined by single-crystal X-ray diffraction (SC-XRD, Figure 2d). The zwitterionic nature of the product stems from the FLP activation of THF, and is analogous to those reported in the literature for other classic FLP systems (Figure 2a).^7^ This structure confirms the potential of bismuth to engage in FLP-mediated transformations, thus expanding its capabilities into new reactivity territory previously unknown for this element. The fact that Dostál Bi­(I) led to uncontrolled reactivity truly highlights the importance of the ligand architecture of the bismuth LB in tuning reactivity.

**Figure 2:**
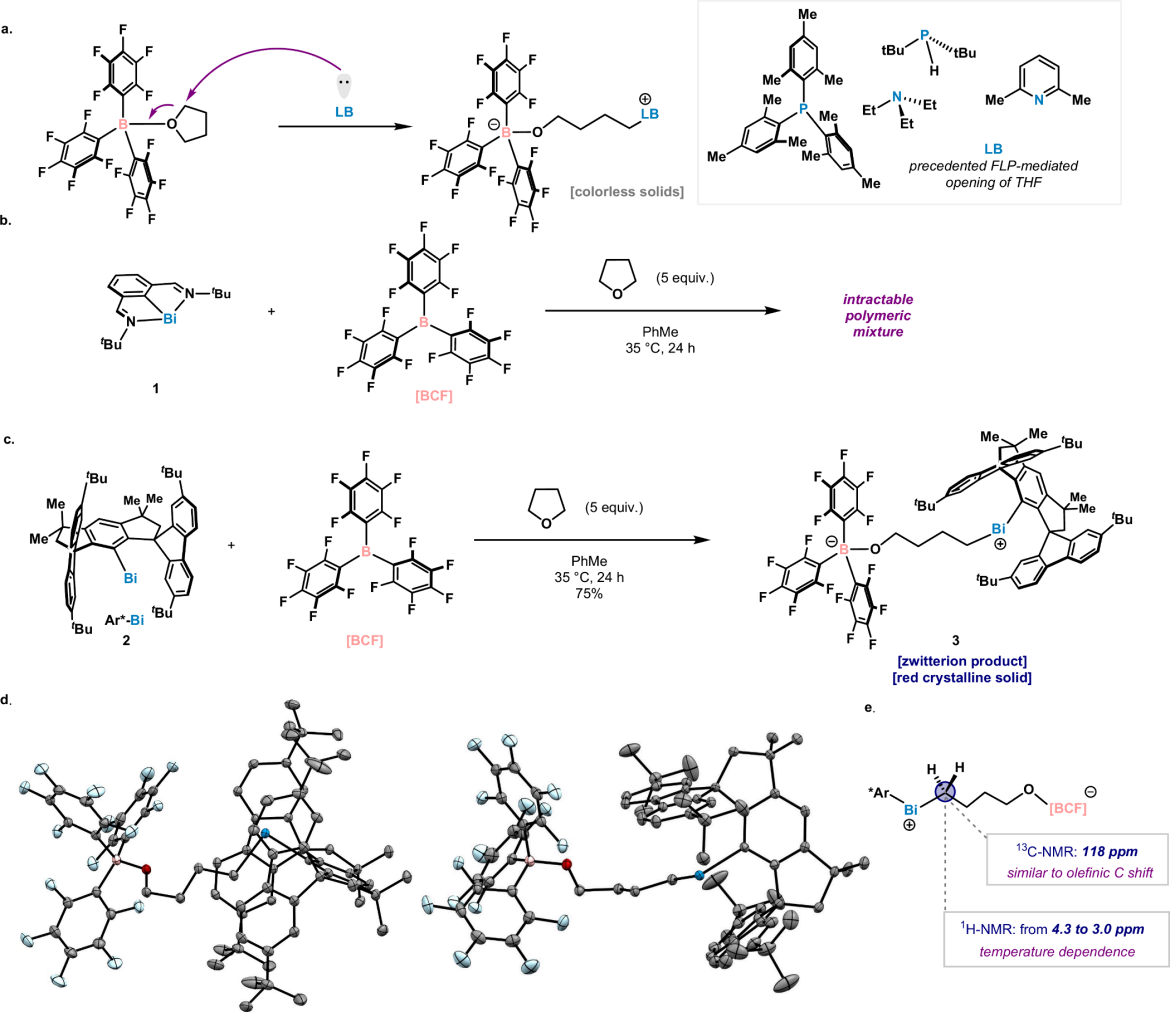
(a) Previously reported ring openings of THF with FLP approach. (b) Attempts toward FLP-mediated opening of THF with **1**. (c) This work: monocoordinated bismuthinidene-based FLP for the activation of tetrahydrofuran. (d) Solid-state structure of **3** at 100 K in two orientations (anisotropic displacement ellipsoids are displayed at a probability level of 50%; hydrogen atoms and disordered solvent molecules are omitted for clarity. (e) Selected NMR features of **3**.

Although the bismuth core in **3** is bonded to two differently hybridized carbon atoms, the two single Bi–C bond lengths are nearly identical: Bi–C­(sp^2^) (2.2509(14) Å) and Bi–C­(sp^3^) (2.2578(15) Å). Remarkably, the Bi–C­(sp^2^) bond is 0.03 Å shorter when compared to bismuth complexes bearing the same ligand scaffold.^6,9^ We postulate that the cationic and highly electron deficient Bi­(III) center promotes a contraction of the atomic orbitals, thus increasing overlap between Bi and C. Interestingly, the methylene group directly bound to bismuth exhibits highly unusual spectroscopic behavior, with the ^13^C signal observed at +118 ppm, while the ^1^H signal shows significant temperature dependence resonating from 4.3 ppm at 50 °C to 3.0 ppm at −70 °C (Figure 2e). At this stage, we cannot rule out that the unusual chemical shift may be attributed to a Spin–Orbit Heavy Atom on Light Atom (SO-HALA) effect caused by the Bi center, intrinsic ring current effects from the fluorene arms, or a combination of both.^10^

Previously reported zwitterionic compounds derived from the activation of THF by classic FLP pairs are typically colorless (Figure 2a);^7^ yet, compound **3** features an intense red color, which is confirmed by UV–vis spectroscopy (Figure 3a).

**Figure 3:**
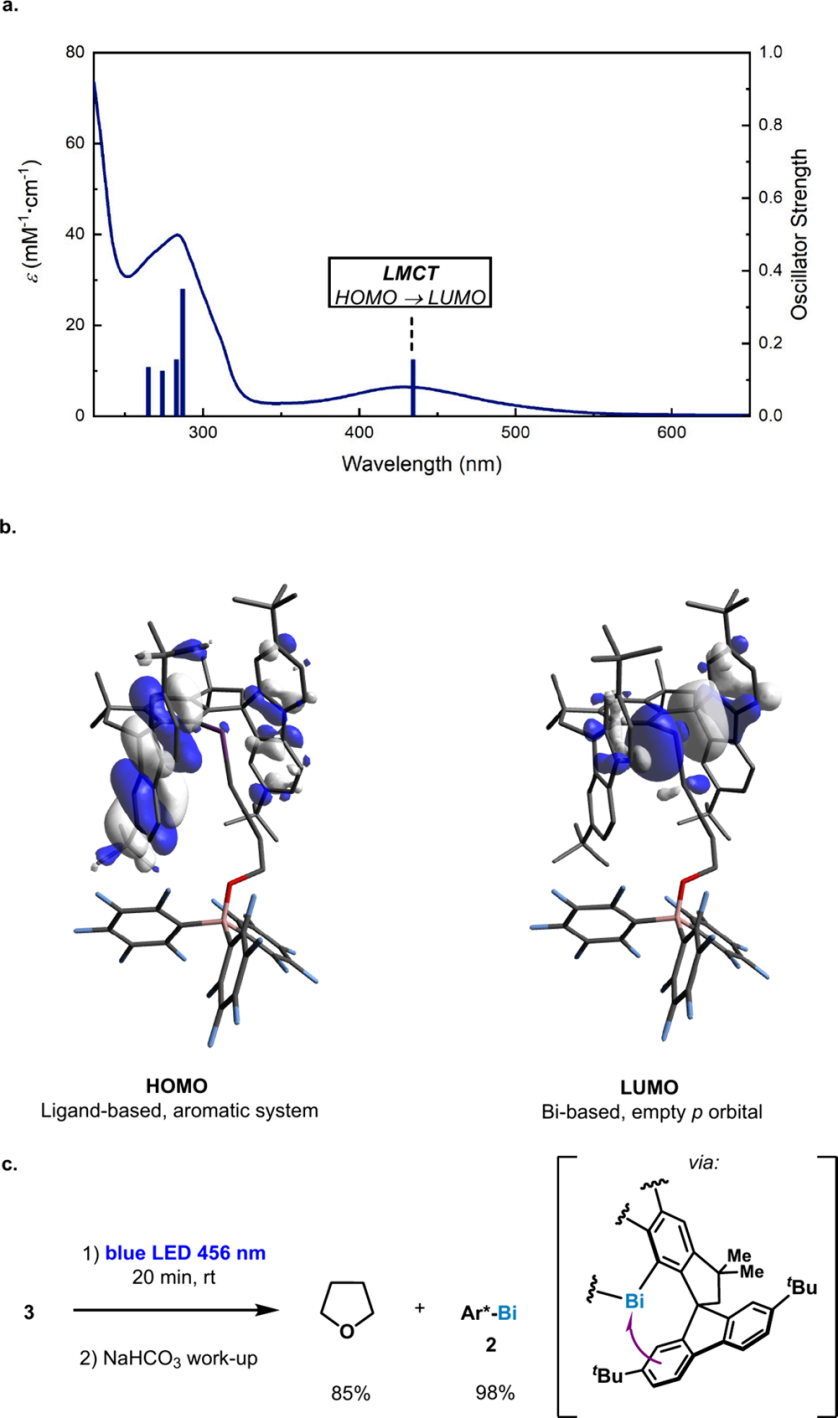
(a) Solid blue line: experimental UV–vis spectrum of a 1.3 mM solution of **3** in CH_2_Cl_2_; Blue bars: TD-DFT excited transitions. (b) TD-DFT: HOMO and LUMO of **3**. (c) Photomediated ring closing.

The measured absorption spectrum of **3** exhibits a single band in the visible region, with λ_max_ centered at 430 nm. TD-DFT calculations attribute the latter absorption band to a ligand-to-metal-charge-transfer event (LMCT) corresponding to a HOMO-to-LUMO transition. The HOMO is localized on the aromatic ring of the ^*t*^BuMsFluind scaffold, while the LUMO is predominantly localized on an empty 6*p* orbital at the bismuth center (see Supporting Information Section 4 for comprehensive computational analysis). While previously reported bismuth complexes in our group possess absorption features in the visible region enabled by MLCT^11^ or LLCT^12^ transitions, here we report an LMCT process occurring between the 12π electrons system on the fluorene and the cationic Bi­(III) center. This unique activity highlights the myriad of possibilities that bismuth can offer by rational ligand design. Indeed, we were able to exploit this LMCT event to promote the reversible ring closing of C­(sp^3^)–O bond in **3**. When **3** is irradiated with blue LEDs for 20 min at 25 °C, THF could be obtained in 85% yield as determined by NMR spectroscopy. Even more notable is the fact that if the reaction mixture is diluted and quenched with NaHCO_3_ under argon (to trap the BCF product), Bi­(I) **2** is recovered in 98% NMR yield,^13^ implying that monocoordinated complex **2** acts as an excellent nucleofuge in a 5-*exo*-*tet*-type reaction.^14^

In summary, we have demonstrated a successful synthetic application of a low-valent Bi­(I) complex as a Lewis base in the ring-opening of THF, in combination with BCF, thus adding Bi to the arsenal of pnictogens used in this type of FLP reactivity. The synergistic interplay between the bulky monocoordinated Bi­(I) complex **2** and the BCF Lewis acid enables the efficient activation of tetrahydrofuran, yielding a zwitterionic product with distinctive spectroscopic properties. Among them, it is remarkable to highlight the unprecedented LMCT band at 430 nm, originating from the flanking π-system to the empty 6*p* of the Bi center. The unique electronic and steric features of the bismuth center, in combination with its carefully designed ligand architecture, underscore its potential to expand the reactivity scope of FLP systems beyond traditional nitrogen- or phosphorus-based Lewis bases. These findings truly open new avenues for the development of transition-metal-free strategies in small molecule activation and organic synthesis. This initial communication serves as the cornerstone for ongoing investigations in our laboratory aimed toward exploiting bismuth­(I)-based FLPs in catalysis, thus paving the way for innovative applications in organometallic chemistry.

## Supplementary Material





## Notes

General considerations, synthetic procedures, elemental analysis, NMR spectra, and crystallographic information (PDF)
Cartesian coordinates for **3** (XYZ)
