# Supplementary material for: Flash Communication: Bismuthinidenes as Lewis Bases in Reversible C(sp3)–O Bond Cleavage
Source: Organometallics. 2025 Aug 20;44(17):1888–92. doi: 10.1021/acs.organomet.5c00260 (PMC12421677; doi:10.1021/acs.organomet.5c00260)
Supplement: Supplementary file 1 [file om5c00260_si_001.pdf]

Flash Communication: Bismuthinidenes as Lewis Bases in Reversible $C(sp^3)$-O Bond Cleavage

Davide Spinnato,[a] Hye Won Moon,[a] Markus Leutzsch,[a] Nils Nöthling[a] and Josep Cornella[a]*

[a]*Max-Planck-Institut für Kohlenforschung, Kaiser-Wilhelm-Platz 1, 45470, Mülheim an der Ruhr, Germany.*

Correspondence to: cornella@kofo.mpg.de

Supporting Information

1. General considerations......**S3**
2. Synthesis and Characterization of 3......**S4**
2.1. Synthesis of **3**...... S4
2.2. Characterization of **3**...... S4
3. Cyclic voltammetry of 3......**S11**
4. UV-vis and TD-DFT of 3......**S11**
5. Reactivity of 3: photo-mediated ring closing......**S13**
6. X-ray Crystal Structure Analysis......**S14**
6.1. Single crystal structure analysis of **3**...... S14
7. References......**S27**

1. General considerations

All experiments were conducted in flame-dried glassware under argon atmosphere by using standard Schlenk techniques or a MBraun glovebox and freshly dried and degassed solvents. (THF, THF-d_8, benzene-d_6 and *n*-pentane were distilled from potassium and stored over 4 Å molecular sieves (activated at 200 °C under a high vacuum for three days) under argon prior to use. Anhydrous $BiBr_3$ (97%) was purchased from Strem Chemicals, Cobaltocene (min. 98%) were purchased from Sigma Aldrich, while Tris(pentafluorophenyl)borane (purity > 97%) was purchased from TCI chemicals and were stored in the freezer of an argon-filled glovebox prior to use. ^{t}Bu-MsFluind-Bi(I) (**1**) was synthesized according to the reported procedure.[1] All other reagents were obtained from commercial suppliers and used without further purification.

NMR data were recorded using a Bruker AVIII 500 MHz NMR or a Bruker AVIIIHD 300 MHz NMR spectrometer. ^{1}H and ^{13}C chemical shifts are reported relative to the solvent residual peaks as an internal reference. For ^{1}H NMR the following residual proton peaks of the deuterated solvents were used: THF-d_8, δ_H δ 1.72 ppm. For ^{13}C NMR: THF-d_8, δ 25.31 ppm. Unless mentioned otherwise ^{13}C and ^{11}B spectra were generally acquired with broadband proton decoupling. Other nuclei (^{19}F, ^{11}B) are reported relative to common standards ($CFCl_3$, $BF_3{\cdot}OEt_2$,). ESI-MS: ESQ 3000 (Bruker). The details of single crystal X-ray diffraction analysis, and NMR studies are described in the following sections.

2. Synthesis and Characterization of 3

2.1. Synthesis of **3**

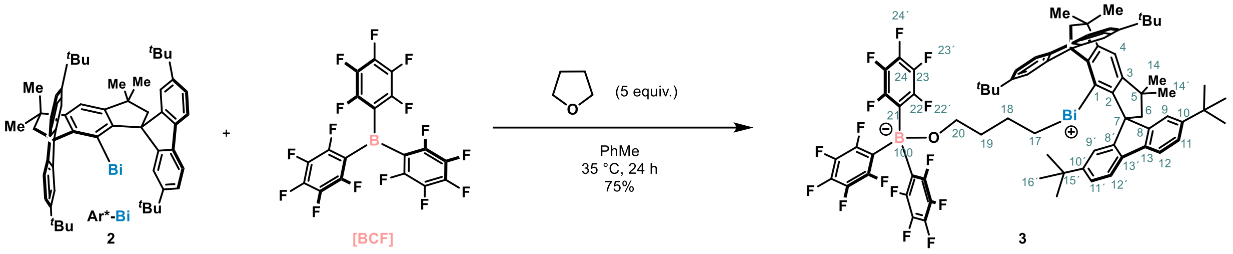

Procedure: In an argon-filled glove box, BCF (25.6 mg, 0.0500 mmol, 1.0 equiv.) was weighted in a 10 mL oven-dried Schlenk tube, 1.5 mL of toluene (anhydrous) was added, followed by the addition of THF (20 µL, 0.25 mml, 5.0 equiv.). Finally, **2** (47 mg, 0.0500 mmol, 1.0 equiv.) was added and the reaction mixture was stirred at 35 °C under Ar outside the glovebox. After 24 h, a bright red solution is formed. Then, the solvent is partially evaporated and the desired product is precipitated by the addition of 10 mL of pentane. The heterogeneous mixture is transferred to a glass-fritted filter (porosity IV) and the solid material is washed several times with anhydrous pentane (total amount 15 mL) under Ar. The bright red solid is dried under high vacuum to obtain **3** (58 mg, 75%).

2.2. Characterization of **3**

^{1}H NMR (500 MHz, THF-*d$_8$*, 203 K): δ 8.15 (s, 1H, H4), 7.89 (d, *J* = 8.1 Hz, 2H, H12), 7.69 (d, *J* = 7.9 Hz, 2H, H12'), 7.62 (d, *J* = 8.1 Hz, 2H, H11), 7.56 (s, 2H, H9), 7.51 (d, *J* = 8.0 Hz, 2H, H11'), 7.32 (s, 2H, H9'), 3.21 (s, 2H, H20), 3.09 – 2.99 (m, 2H, H18), 2.63 (d, *J* = 14.3 Hz, 2H, H6a), 2.47 (d, *J* = 14.3 Hz, 2H, H6b), 1.80 (s, 6H, H14), 1.66 (s, 6H, H14'), 1.42 – 1.34 (m, 2H, H17), 1.26 (s, 18H, H16), 1.18 (s, 18H, H16'), 0.55 (bs, 2H, H19).

^{13}C NMR (126 MHz, THF-*d$_8$*, 203 K): δ 225.7 (C1), 161.1 (C2), 160.1 (C3), 156.6 (C10), 154.6 (C10'), 154.1 (C8'), 152.9 (C8), 148.8 (dm, *J* = 239.7 Hz, C22), 138.9 (dm, *J* = 226.3 Hz, C24), 137.0 (dm, *J* = 237.4 Hz, C23), 134.1 (C13), 132.3 (C13'), 130.4 (C11), 126.9 (C11'), 124.7 (C9), 123.7 (C12), 122.1 (C9' & C12'), 118.4 (C4), 118.0 (C17), 66.6 (C20), 64.9 (C7), 51.8 (C6), 49.6 (C19), 47.0 (C5), 36.2 (C15), 35.6 (C15'), 33.5 (C14'), 32.8 (C14), 31.7 (C16'), 31.5 (C16), 17.9 (C18). *The signal of C21 was not found.

^{19}F NMR (470 MHz, THF-*d$_8$*, 203 K): δ -133.65 (d, *J* = 25.0 Hz, 6F, 22'), -164.41 (t, *J* = 20.4 Hz, 3F, 24'), -167.60 (app. t, *J* = 22.0 Hz, 6F, 23').

^{11}B NMR (160 MHz, THF-*d$_8$*, 203 K): δ –2.9.

HRMS (ESIpos): calc'd for $C_{78}H_{73}B_1Bi_1F_{15}O_1^+$ [Na]$^+$ 1553.52079; found 1553.52112.

XRD: single crystals of **3** suitable for X-ray diffraction analysis were obtained by layering pentane into a filtered THF solution of **3**.

Stability: air sensitive.

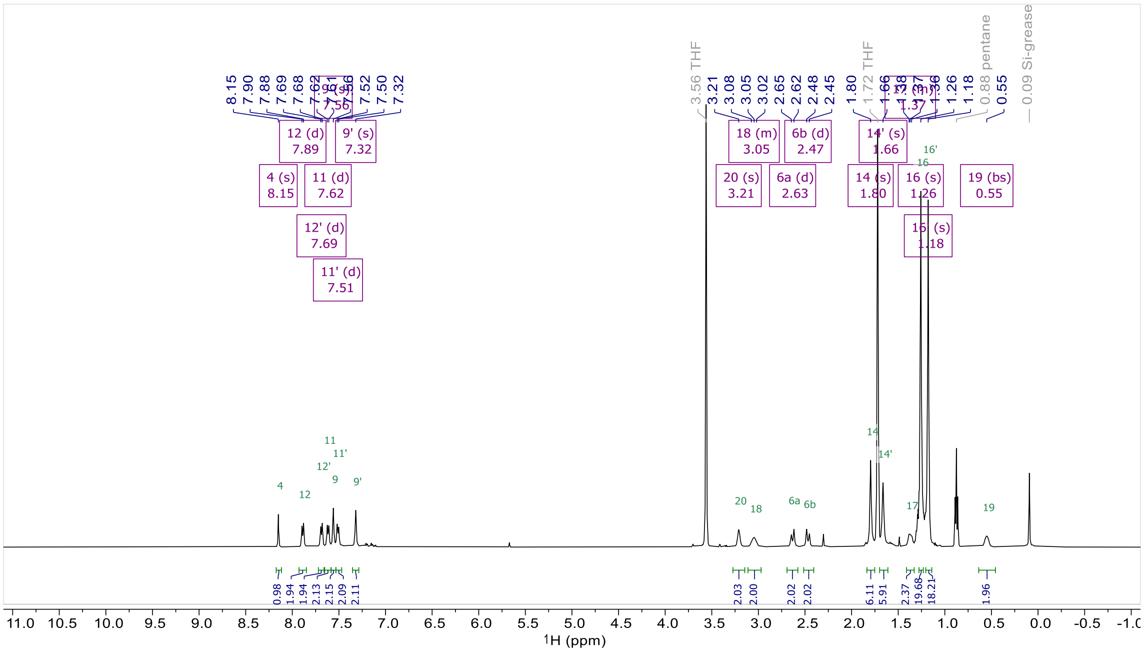

Figure S1. ^{1}H NMR spectrum of **3** (500 MHz, THF-d_8, 203 K).

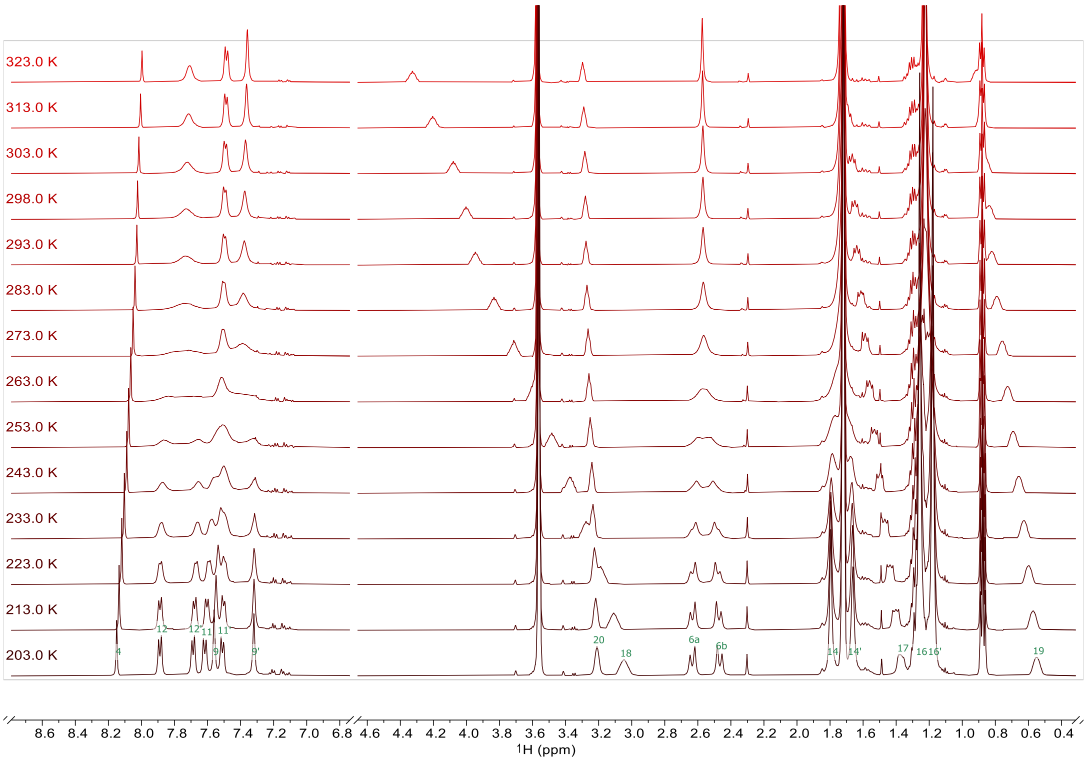

Figure S2. Stacked Variable Temperature (VT) ^{1}H NMR spectra of **3** (500 MHz, THF-d_8).

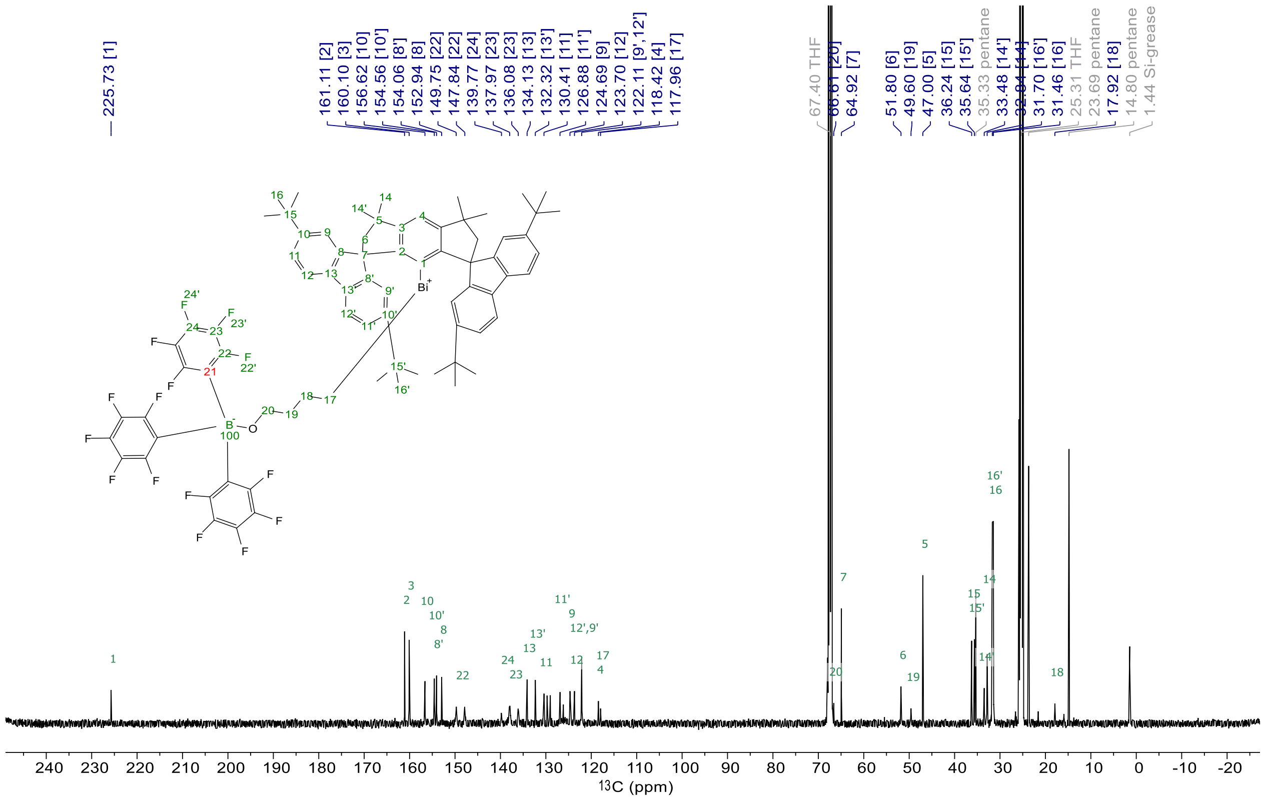

Figure S3. ^{13}C NMR spectrum of **3** (126 MHz, THF-d_8, 203 K).

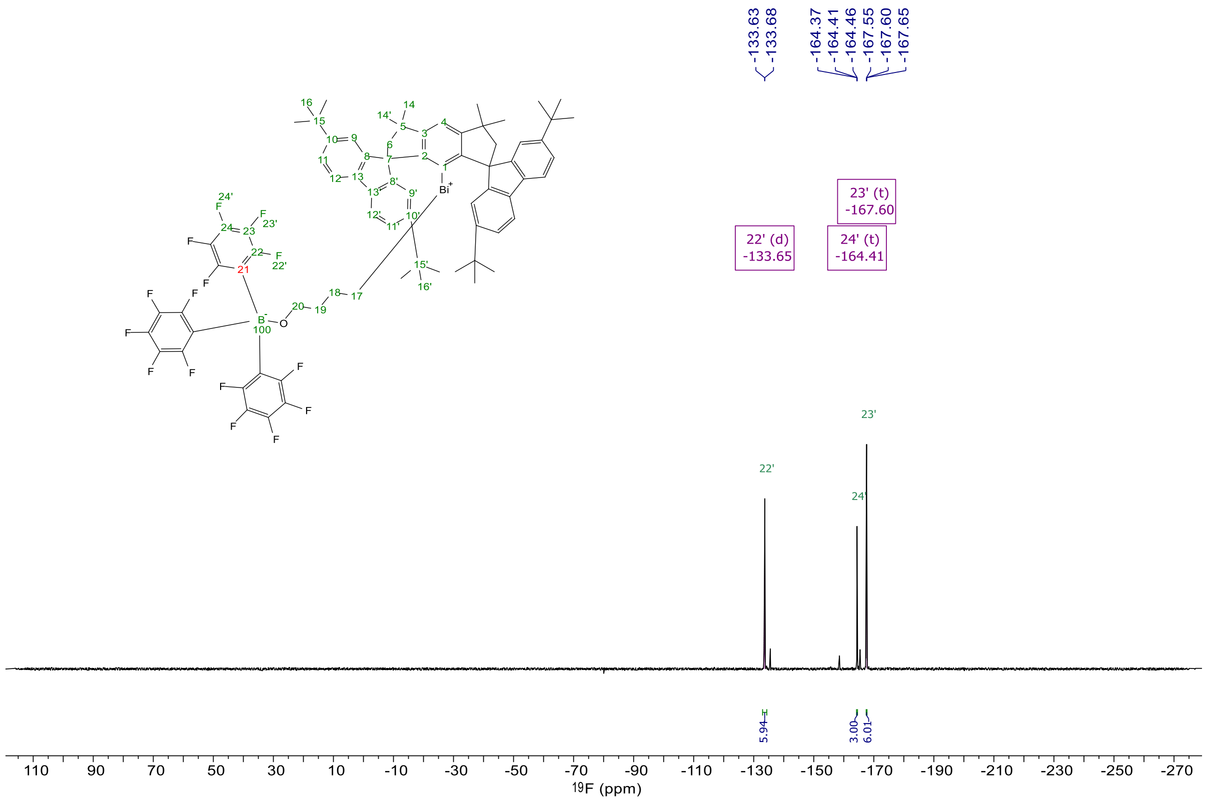

Figure S4. ^{19}F NMR spectrum of **3** (470 MHz, THF-d_8, 203 K).

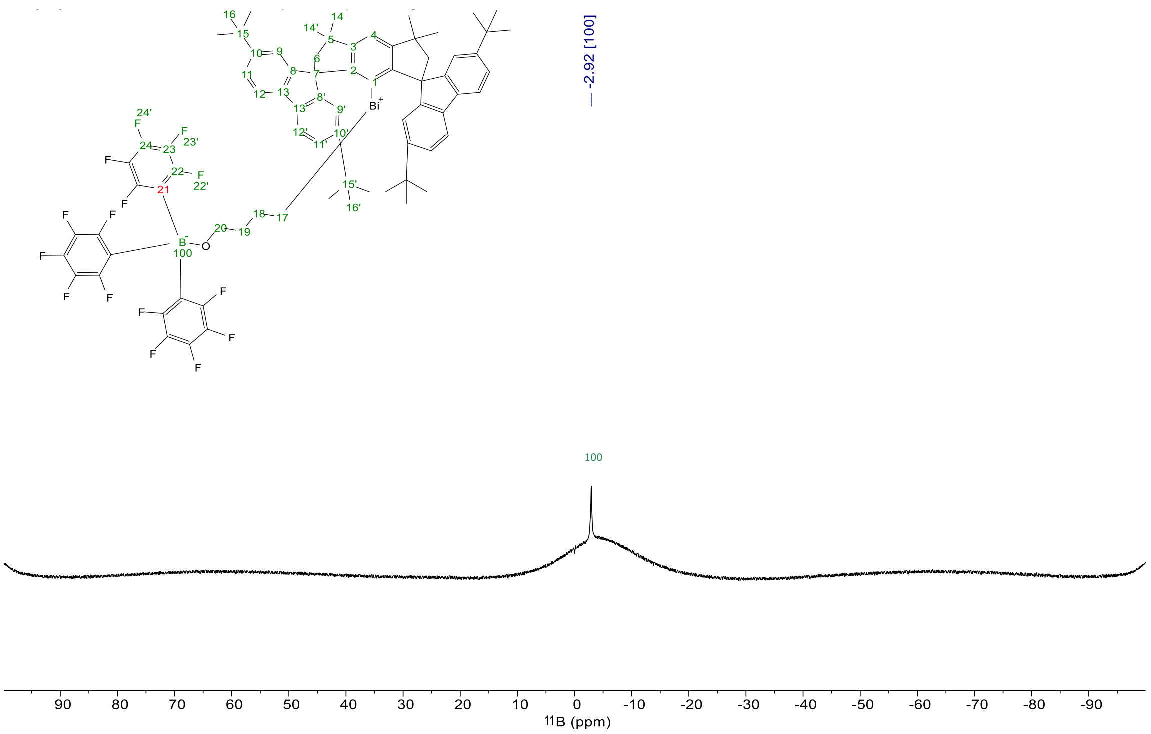

Figure S5. ^{11}B NMR spectrum of **3** (160 MHz, THF-d_8, 203 K).

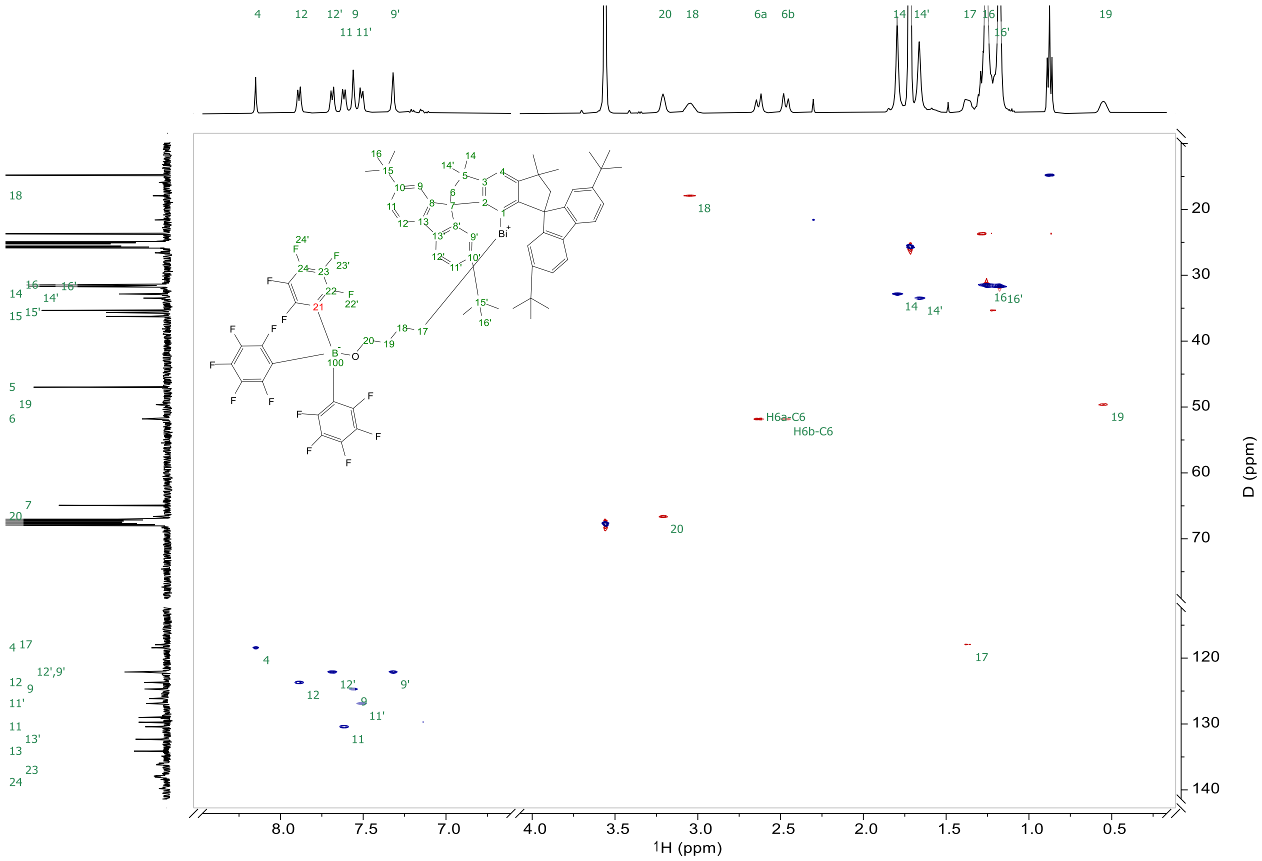

Figure S6. ^{1}H-^{13}C edited HSQC spectrum of **3** (500 MHz, THF-d_8, 203 K).

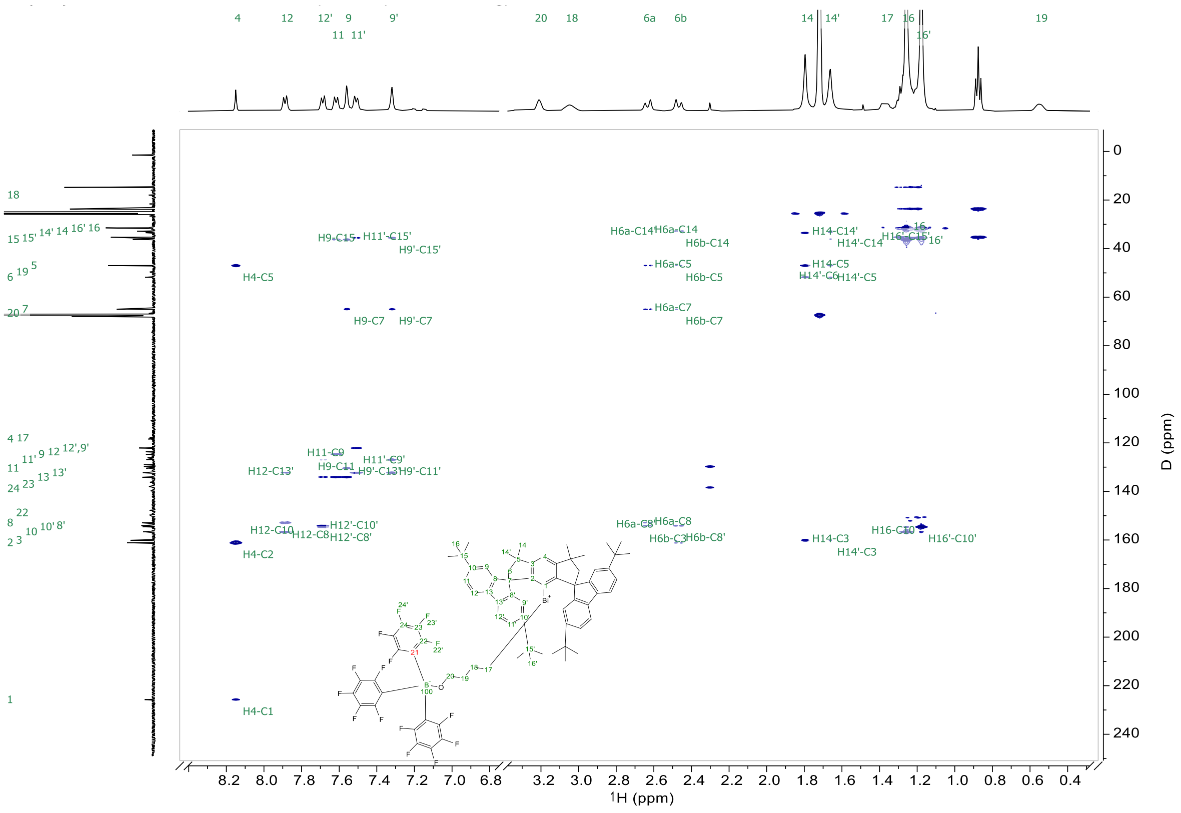

Figure S7. ^1H-^{13}C HMBC spectrum of **3** (500 MHz, THF-d_8, 203 K).

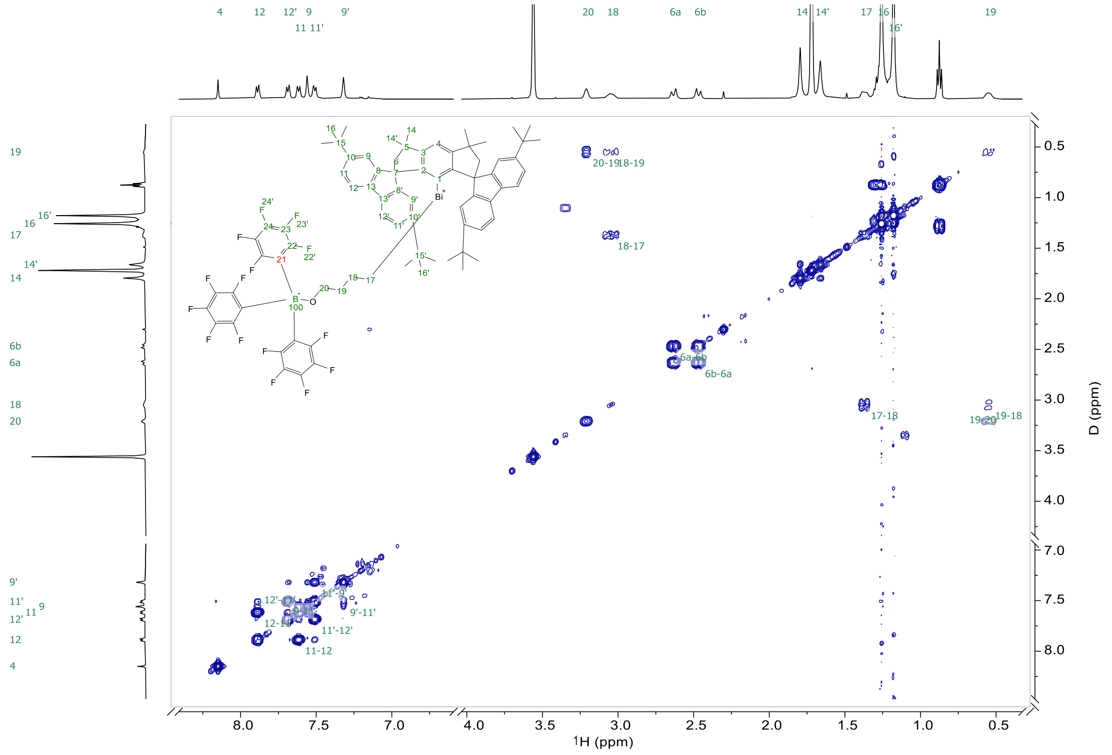

Figure S8. ^1H-^1H COSY spectrum of **3** (500 MHz, THF-d_8, 203 K).

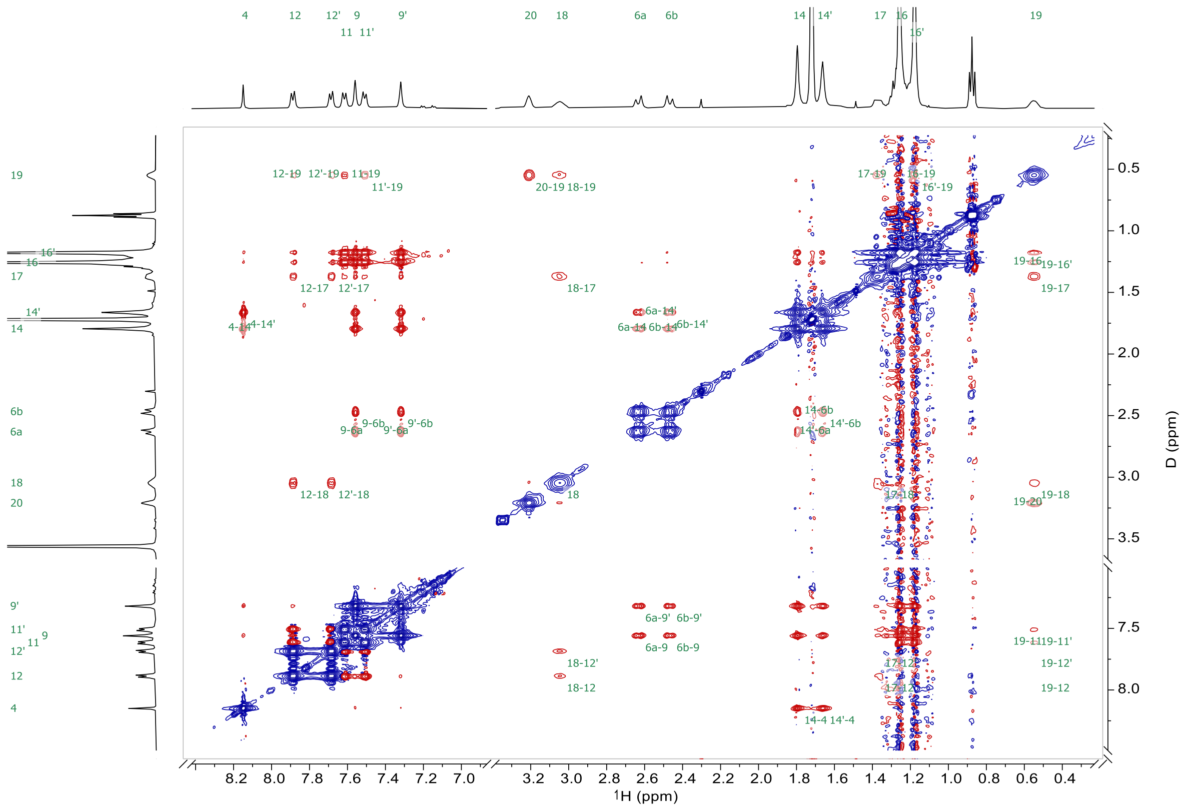

Figure S9. ^{1}H-^{1}H ROESY spectrum of **3** (500 MHz, THF-d_8, 203 K).

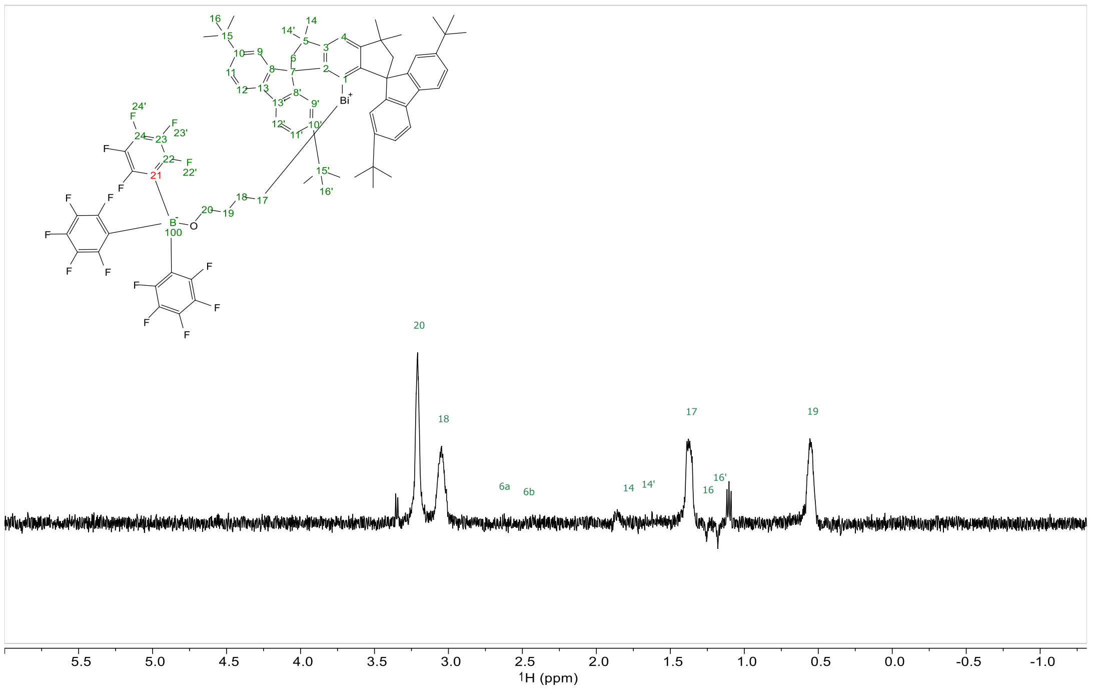

Figure S10. ^{1}H NMR selective TOCSY spectrum from excitation of H-20 **3** (500 MHz, THF-d_2, 203 K).

3. Cyclic voltammetry of 3

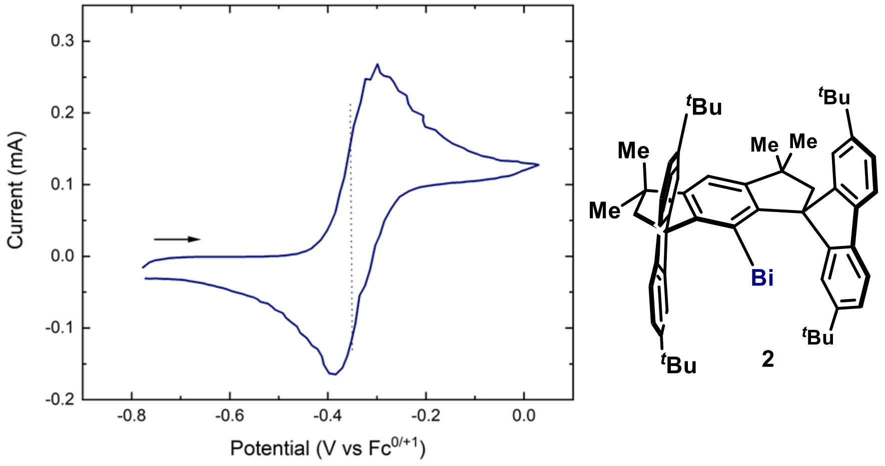

Figure S11. Cyclic voltammogram of **3** (cathodic scan) in dichloromethane using 0.1 M [nBu_4N][BArF] as supporting electrolyte at ambient temperature; scan rate: 50 mV/s, referenced to $Fc^{0/+}$.

4. UV-vis and TD-DFT of 3

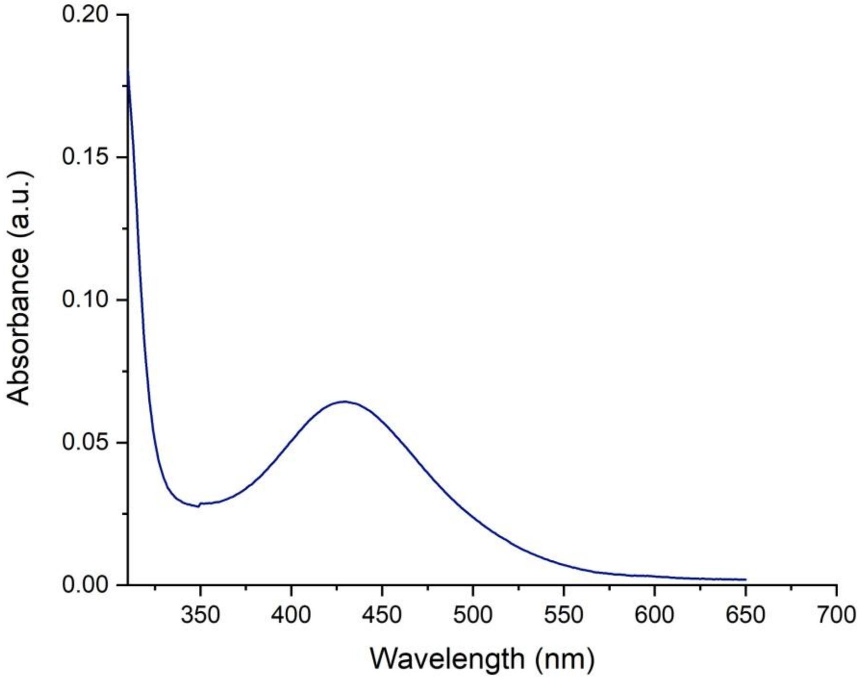

Figure S12. UV-Vis absorption spectra of **3** (1.3 mM in CH_2Cl_2, 298K), wavelength range 300-700nm.

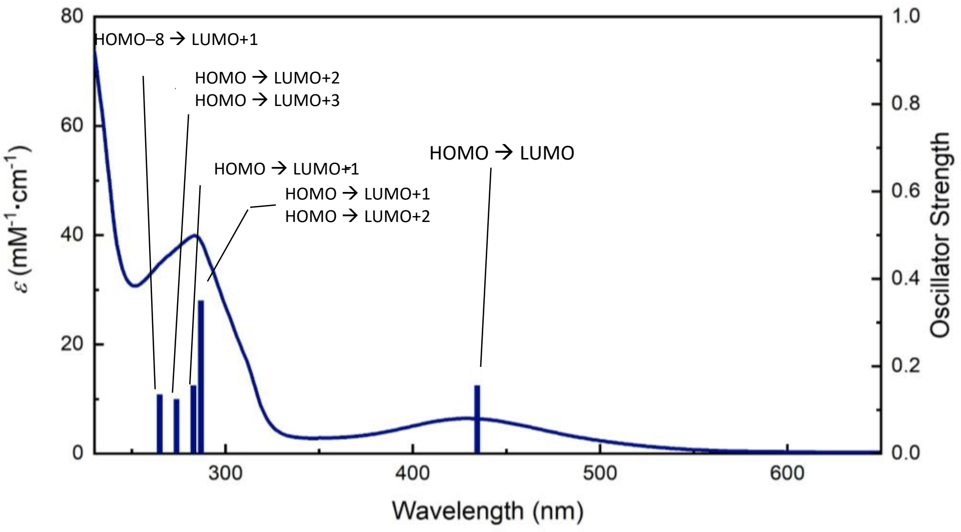

Figure S13. Experimental UV-vis spectrum for **3** (blue line) super-imposed with TD-DFT excited transitions (blue bars) at the PBE0-D3BJ/def2-TZVP/CPCM(THF) level of theory with Def2-ECP (Bi)

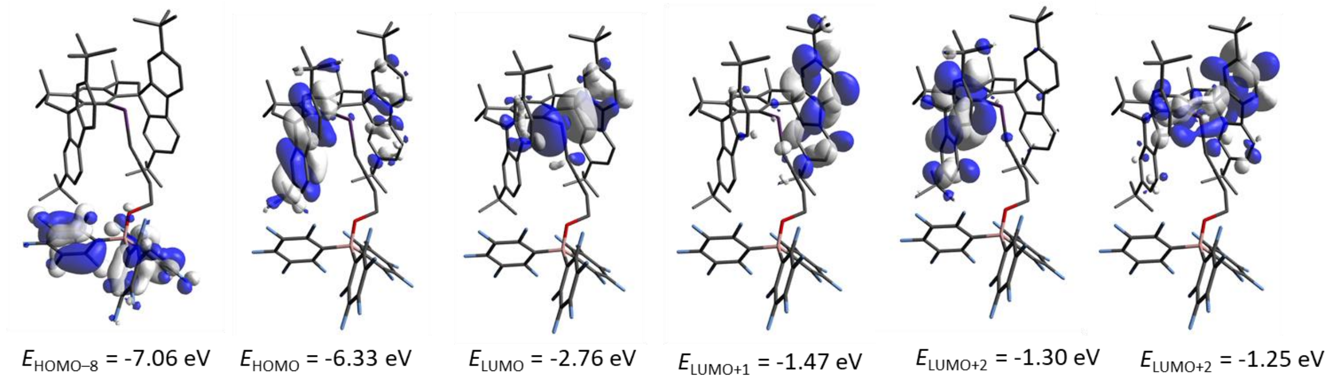

Figure S14. Selected molecular orbital isosurfaces and orbital energies for **3**. Hydrogen atoms are omitted for clarity.

5. Reactivity of 3: photo-mediated ring closing

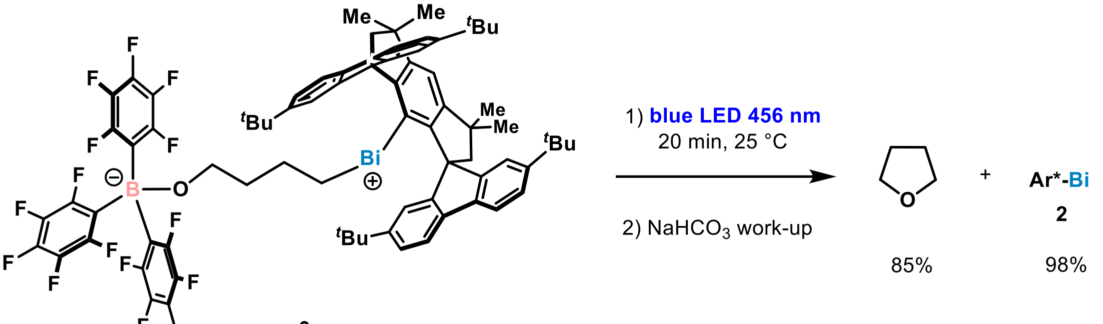

Procedure: In an argon-filled glove box, **3** (7.50 mg, 0.00500 mmol, 1 equiv.) was placed in an oven-dried culture tube, followed by benzene-d_6 (0.30 mL, 16 mM) and the reaction vial was sealed with a screw cap containing a Teflon™-coated rubber septum. The heterogeneous mixture was placed in the irradiation set-up (2 × Kessil lamp, 456 nm, 100% intensity) outside of the glovebox. After 20 min, the culture tube is brought inside the glovebox, the reaction mixture is diluted with 0.4 mL of benzene-d_6 and $NaHCO_3$ (4.10 mg, 0.0500 mmol, 10 equiv.) and left stirring for 5 min. Finally, trichloroethylene (4.40 µL, 0.0500 mmol, 10 equiv.) is added as internal standard and the homogeneous solution is added into an NMR tube. ^{1}H-NMR indicates formation of **2** in 98% NMR yield and THF in 85% NMR yield.

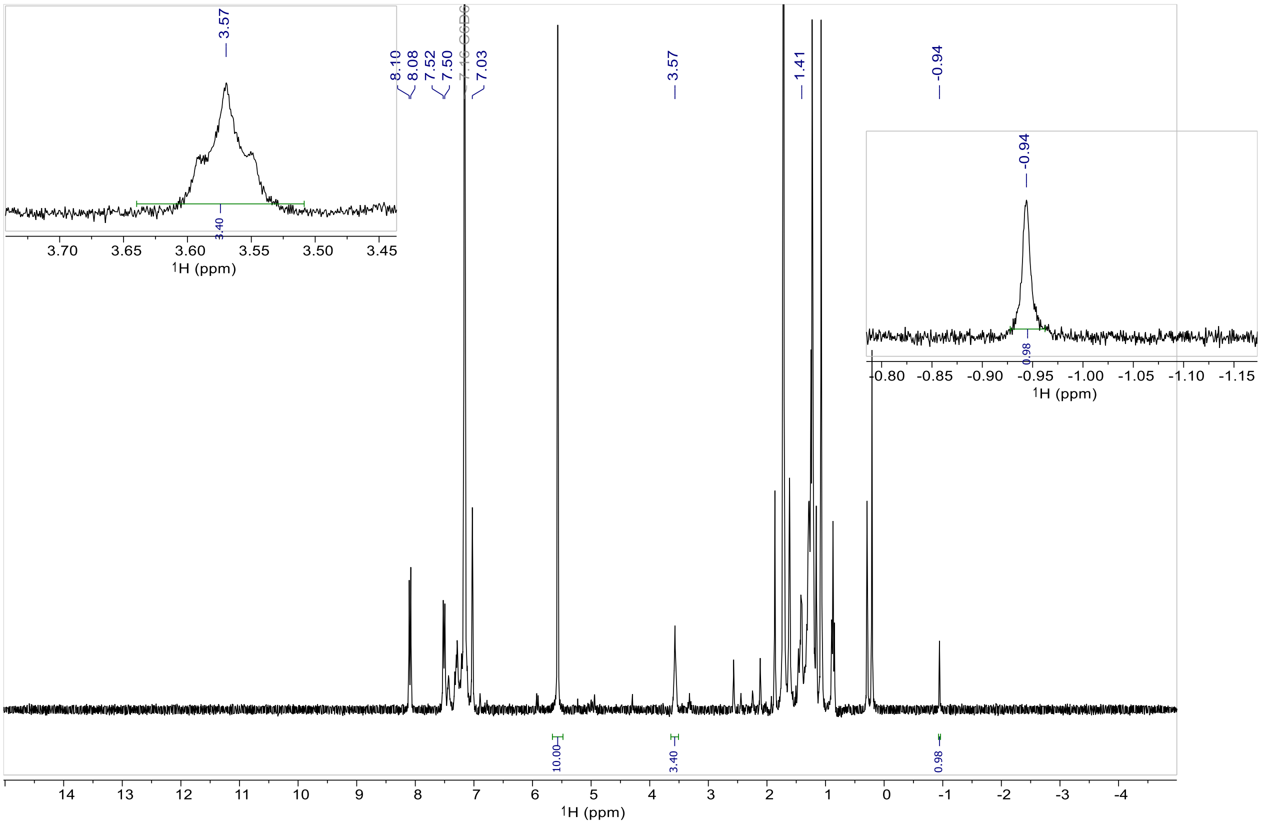

Figure S15. ^{1}H NMR spectrum (300 MHz, 298 K) of crude reaction mixture of the photo-mediated ring closing starting from **3** (the peak at –0.98 ppm is the characteristic peak for **2**)[1].

It is important to add $NaHCO_3$ to quench free BCF after the reaction in order to recover **2**. When the mixture is not quenched with base, by-products are formed. One of the detectable by-products is a trimetallic bismuth(I)-based allyl cation which is formed due to the presence of trace amounts of moisture in the mixture.[2]

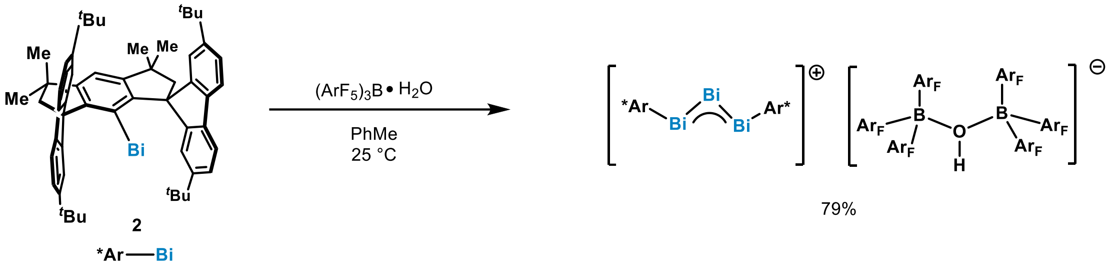

6. X-ray Crystal Structure Analysis

6.1. Single crystal structure analysis of **3**

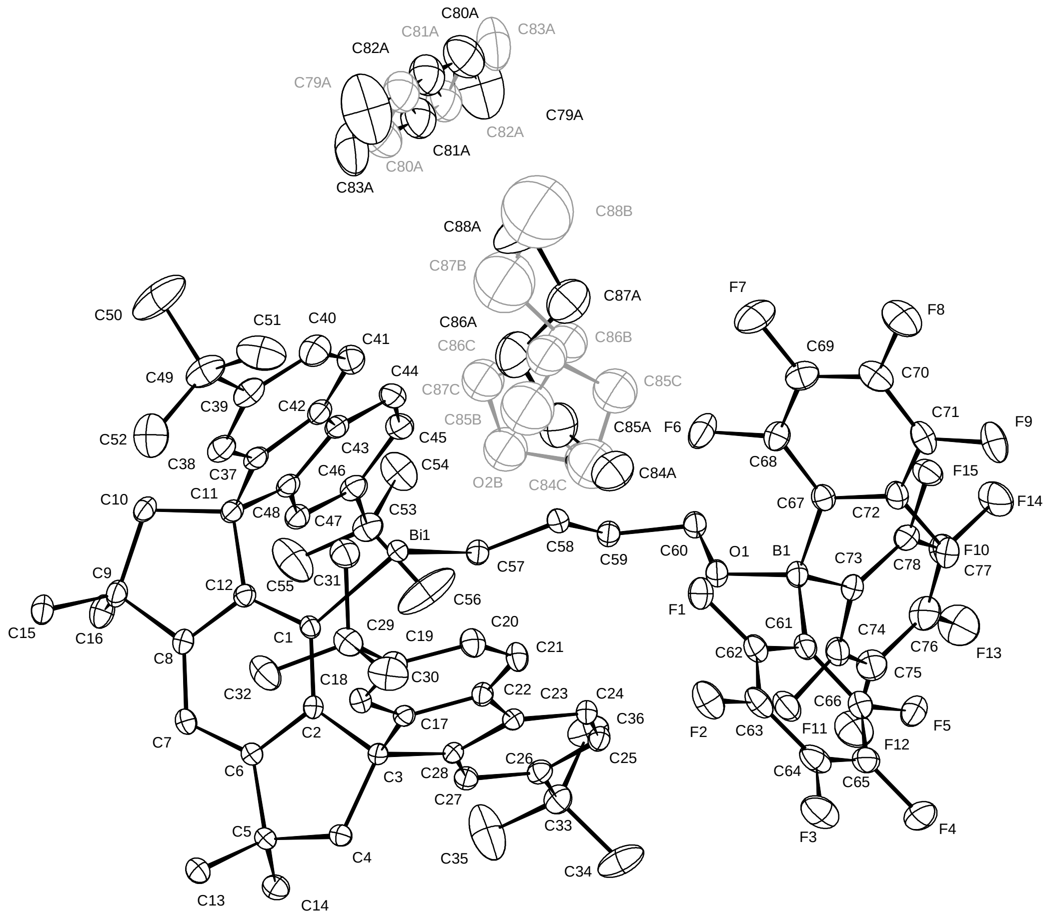

Figure S16. The molecular structure of **3**. H atoms have been removed for clarity. Main structure shown in black and disordered parts shown in grey.

X-ray Crystal Structure Analysis of 3:

$C_{85.25}$ H_{90} B Bi F_{15} $O_{1.25}$, M_r = 1639.36 g mol^{-1}, orange prism, crystal size 0.206 x 0.148 x 0.12 mm^3, Triclinic, space group *P*-1 [2], a = 14.8855(12) Å, b = 16.9947(13) Å, *c* = 17.1541(14) Å, α= 74.154(4)°, β= 67.280(4)°, γ = 77.962(4)°, *V* = 3824.6(5) Å^3, *T* = 100(2) K, *Z* = 2, D_{calc} = 1.424 g·cm^3, λ = 0.71073 Å, $\mu(Mo\text{-}K_\alpha)$ = 2.387 mm^{-1}, Gaussian absorption correction ($T_{\min}$ = 0.7009, $T_{\max}$ = 0.8467), Bruker-AXS D8 Venture with Photon III detector and IµS Diamond microfocus Mo-anode X-ray source, 2.003 < θ < 30.999°, 527185 measured reflections, 24373 independent reflections, 22240 reflections with $I > 2\sigma(I)$, R_{int} = 0.0964. The structure was solved by *SHELXT* and refined by full-matrix least-squares (*SHELXL*) against F^2 to R_1 = 0.0219 [$I > 2\sigma(I)$], wR_2 = 0.0530 [all data], 1017 parameters and 81 restraints.

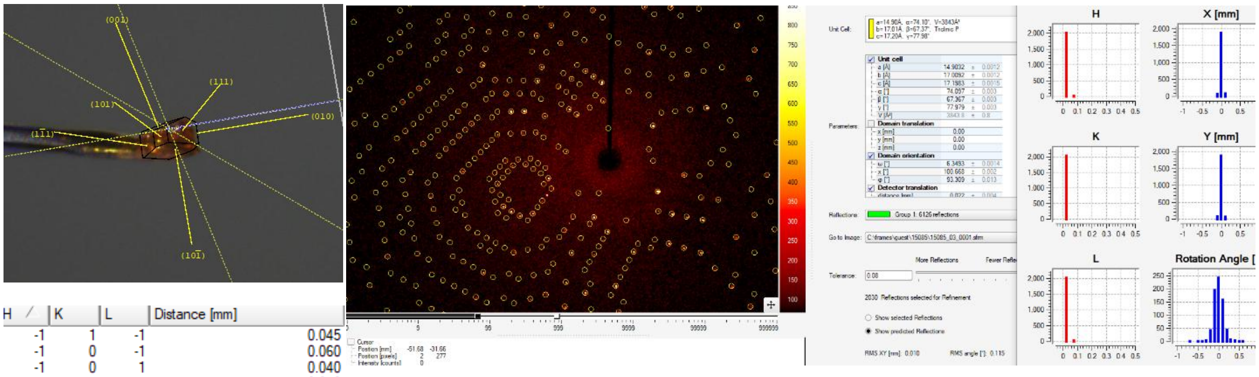

H	K	L	Distance [mm]
-1	1	-1	0.045
-1	0	-1	0.060
-1	0	1	0.040
-1	1	1	0.030
0	0	1	0.015
0	0	-1	0.075
0	1	0	0.075
0	-1	0	0.080
0	-1	-2	0.080
1	-1	1	0.060
1	0	1	0.055
1	1	1	0.050
1	0	-1	0.070

Figure S17. Crystal faces and unit cell determination/refinement of compound **3**.

INTENSITY STATISTICS FOR DATASET

Resolution	#Data	#Theory	%Complete	Redundancy	Mean I	Mean I/s	Rmerge	Rsigma
Inf - 2.74	771	798	96.6	11.53	90.97	42.60	0.0417	0.0382
2.74 - 1.83	1808	1812	99.8	15.24	62.14	47.86	0.0445	0.0206
1.83 - 1.46	2536	2536	100.0	16.38	43.36	43.85	0.0517	0.0180
1.46 - 1.27	2672	2672	100.0	15.71	32.17	37.20	0.0672	0.0213
1.27 - 1.16	2462	2462	100.0	15.71	26.70	32.94	0.0790	0.0241
1.16 - 1.07	2772	2772	100.0	13.33	24.15	28.11	0.0847	0.0284
1.07 - 1.01	2500	2500	100.0	12.14	20.17	23.91	0.0966	0.0337
1.01 - 0.96	2572	2572	100.0	11.47	16.65	20.60	0.1113	0.0397
0.96 - 0.92	2476	2476	100.0	10.78	14.91	18.50	0.1234	0.0456
0.92 - 0.88	2898	2898	100.0	10.38	13.03	16.53	0.1340	0.0515
0.88 - 0.85	2542	2542	100.0	10.10	12.29	15.32	0.1437	0.0558
0.85 - 0.82	3020	3020	100.0	9.42	10.30	12.84	0.1614	0.0659
0.82 - 0.80	2242	2242	100.0	9.24	9.61	12.16	0.1702	0.0706
0.80 - 0.78	2406	2406	100.0	9.02	8.91	11.28	0.1796	0.0765
0.78 - 0.76	2812	2812	100.0	8.79	8.33	10.54	0.1940	0.0829
0.76 - 0.74	2932	2932	100.0	8.10	7.62	9.16	0.2063	0.0938
0.74 - 0.73	1750	1750	100.0	7.95	7.00	8.45	0.2214	0.1020
0.73 - 0.71	3512	3512	100.0	7.55	6.58	7.71	0.2223	0.1105
0.71 - 0.70	2010	2010	100.0	7.30	6.26	7.25	0.2251	0.1193
0.70 - 0.69	2040	2040	100.0	7.11	6.04	6.91	0.2274	0.1252
0.69 - 0.68	2203	2540	86.7	5.65	5.18	5.88	0.2449	0.1544
0.78 - 0.68	17259	17596	98.1	7.53	6.80	8.11	0.2154	0.1077
Inf - 0.68	50936	51304	99.3	10.56	17.70	19.00	0.0936	0.0432

Complete .cif-data of the compound are available under the CCDC number CCDC-**2470580**.
A resolution cut off (SHEL 999 0.73) was applied to data set to exclude incomplete shells at higher diffraction angles. The solute was modelled using the 'pentane' and 'THF' fragments from the DSR tool, which is available as an Olex2 plugin.[3-5]

Table 1. Crystal data and structure refinement of 3.

Identification code	15085	
Empirical formula	$C_{85.25}$ H_{90} B Bi F_{15} $O_{1.25}$	
Color	orange	
Formula weight	1639.36 g·mol^{-1}	
Temperature	100(2) K	
Wavelength	0.71073 Å	
Crystal system	Triclinic	
Space group	*P*-1, (no. 2)	
Unit cell dimensions	a = 14.8855(12) Å	α= 74.154(4)°.
	b = 16.9947(13) Å	β= 67.280(4)°.
	c = 17.1541(14) Å	γ = 77.962(4)°.
Volume	3824.6(5) Å^3	
Z	2	

Density (calculated)	1.424 $Mg \cdot m^{-3}$
Absorption coefficient	2.387 mm^{-1}
F(000)	1669 e
Crystal size	0.206 x 0.148 x 0.12 mm^3
θ range for data collection	2.003 to 30.999°.
Index ranges	$-21 \le h \le 21$, $-24 \le k \le 24$, $-24 \le l \le 24$
Reflections collected	527185
Independent reflections	24373 [R_{int} = 0.0964]
Reflections with I>2σ(I)	22240
Completeness to θ = 25.242°	99.9 %
Absorption correction	Numerical
Max. and min. transmission	0.8467 and 0.7009
Refinement method	Full-matrix least-squares on F^2
Data / restraints / parameters	24373 / 81 / 1017
Goodness-of-fit on F^2	1.038
Final R indices [I>2σ(I)]	R_1 = 0.0219 wR^2 = 0.0512
R indices (all data)	R_1 = 0.0265 wR^2 = 0.0530
Extinction coefficient	n/a
Largest diff. peak and hole	0.648 and -0.398 $e \cdot Å^{-3}$

Table 2. Bond lengths [Å] and angles [°] of 3.

Bi(1)-C(1)	2.2509(14)	Bi(1)-C(57)	2.2578(15)
F(1)-C(62)	1.3534(19)	F(2)-C(63)	1.349(2)
F(3)-C(64)	1.3458(19)	F(4)-C(65)	1.347(2)
F(5)-C(66)	1.350(2)	F(6)-C(68)	1.3489(19)
F(7)-C(69)	1.346(2)	F(8)-C(70)	1.3434(19)
F(9)-C(71)	1.3462(19)	F(10)-C(72)	1.3555(18)
F(11)-C(74)	1.3587(18)	F(12)-C(75)	1.348(2)
F(13)-C(76)	1.343(2)	F(14)-C(77)	1.3489(19)
F(15)-C(78)	1.3511(18)	O(1)-C(60)	1.4187(18)
O(1)-B(1)	1.4538(19)	C(1)-C(2)	1.3933(19)
C(1)-C(12)	1.393(2)	C(2)-C(3)	1.5175(19)
C(2)-C(6)	1.3955(19)	C(3)-C(4)	1.5602(19)
C(3)-C(17)	1.5191(19)	C(3)-C(28)	1.535(2)
C(4)-H(4A)	0.9900	C(4)-H(4B)	0.9900

C(4)-C(5)	1.560(2)	C(5)-C(6)	1.5210(19)
C(5)-C(13)	1.531(2)	C(5)-C(14)	1.535(2)
C(6)-C(7)	1.390(2)	C(7)-H(7)	0.9500
C(7)-C(8)	1.395(2)	C(8)-C(9)	1.518(2)
C(8)-C(12)	1.393(2)	C(9)-C(10)	1.561(2)
C(9)-C(15)	1.535(2)	C(9)-C(16)	1.537(2)
C(10)-H(10A)	0.9900	C(10)-H(10B)	0.9900
C(10)-C(11)	1.558(2)	C(11)-C(12)	1.520(2)
C(11)-C(37)	1.524(2)	C(11)-C(48)	1.527(2)
C(13)-H(13A)	0.9800	C(13)-H(13B)	0.9800
C(13)-H(13C)	0.9800	C(14)-H(14A)	0.9800
C(14)-H(14B)	0.9800	C(14)-H(14C)	0.9800
C(15)-H(15A)	0.9800	C(15)-H(15B)	0.9800
C(15)-H(15C)	0.9800	C(16)-H(16A)	0.9800
C(16)-H(16B)	0.9800	C(16)-H(16C)	0.9800
C(17)-C(18)	1.389(2)	C(17)-C(22)	1.405(2)
C(18)-H(18)	0.9500	C(18)-C(19)	1.398(2)
C(19)-C(20)	1.407(2)	C(19)-C(29)	1.527(2)
C(20)-H(20)	0.9500	C(20)-C(21)	1.384(2)
C(21)-H(21)	0.9500	C(21)-C(22)	1.406(2)
C(22)-C(23)	1.462(2)	C(23)-C(24)	1.394(2)
C(23)-C(28)	1.4010(19)	C(24)-H(24)	0.9500
C(24)-C(25)	1.385(2)	C(25)-H(25)	0.9500
C(25)-C(26)	1.402(2)	C(26)-C(27)	1.400(2)
C(26)-C(33)	1.534(2)	C(27)-H(27)	0.9500
C(27)-C(28)	1.387(2)	C(29)-C(30)	1.532(2)
C(29)-C(31)	1.537(2)	C(29)-C(32)	1.532(2)
C(30)-H(30A)	0.9800	C(30)-H(30B)	0.9800
C(30)-H(30C)	0.9800	C(31)-H(31A)	0.9800
C(31)-H(31B)	0.9800	C(31)-H(31C)	0.9800
C(32)-H(32A)	0.9800	C(32)-H(32B)	0.9800
C(32)-H(32C)	0.9800	C(33)-C(34)	1.526(2)
C(33)-C(35)	1.522(3)	C(33)-C(36)	1.537(2)
C(34)-H(34A)	0.9800	C(34)-H(34B)	0.9800
C(34)-H(34C)	0.9800	C(35)-H(35A)	0.9800
C(35)-H(35B)	0.9800	C(35)-H(35C)	0.9800

C(36)-H(36A)	0.9800	C(36)-H(36B)	0.9800
C(36)-H(36C)	0.9800	C(37)-C(38)	1.391(2)
C(37)-C(42)	1.414(2)	C(38)-H(38)	0.9500
C(38)-C(39)	1.402(2)	C(39)-C(40)	1.403(3)
C(39)-C(49)	1.534(2)	C(40)-H(40)	0.9500
C(40)-C(41)	1.382(2)	C(41)-H(41)	0.9500
C(41)-C(42)	1.401(2)	C(42)-C(43)	1.464(2)
C(43)-C(44)	1.394(2)	C(43)-C(48)	1.398(2)
C(44)-H(44)	0.9500	C(44)-C(45)	1.391(2)
C(45)-H(45)	0.9500	C(45)-C(46)	1.404(2)
C(46)-C(47)	1.399(2)	C(46)-C(53)	1.531(2)
C(47)-H(47)	0.9500	C(47)-C(48)	1.382(2)
C(49)-C(50)	1.543(3)	C(49)-C(51)	1.535(3)
C(49)-C(52)	1.527(3)	C(50)-H(50A)	0.9800
C(50)-H(50B)	0.9800	C(50)-H(50C)	0.9800
C(51)-H(51A)	0.9800	C(51)-H(51B)	0.9800
C(51)-H(51C)	0.9800	C(52)-H(52A)	0.9800
C(52)-H(52B)	0.9800	C(52)-H(52C)	0.9800
C(53)-C(54)	1.528(2)	C(53)-C(55)	1.531(3)
C(53)-C(56)	1.519(3)	C(54)-H(54A)	0.9800
C(54)-H(54B)	0.9800	C(54)-H(54C)	0.9800
C(55)-H(55A)	0.9800	C(55)-H(55B)	0.9800
C(55)-H(55C)	0.9800	C(56)-H(56A)	0.9800
C(56)-H(56B)	0.9800	C(56)-H(56C)	0.9800
C(57)-H(57A)	0.9900	C(57)-H(57B)	0.9900
C(57)-C(58)	1.522(2)	C(58)-H(58A)	0.9900
C(58)-H(58B)	0.9900	C(58)-C(59)	1.536(2)
C(59)-H(59A)	0.9900	C(59)-H(59B)	0.9900
C(59)-C(60)	1.520(2)	C(60)-H(60A)	0.9900
C(60)-H(60B)	0.9900	C(61)-C(62)	1.392(2)
C(61)-C(66)	1.386(2)	C(61)-B(1)	1.670(2)
C(62)-C(63)	1.384(2)	C(63)-C(64)	1.377(3)
C(64)-C(65)	1.373(3)	C(65)-C(66)	1.389(2)
C(67)-C(68)	1.386(2)	C(67)-C(72)	1.388(2)
C(67)-B(1)	1.670(2)	C(68)-C(69)	1.390(2)
C(69)-C(70)	1.371(3)	C(70)-C(71)	1.377(3)

C(71)-C(72)	1.375(2)	C(73)-C(74)	1.392(2)
C(73)-C(78)	1.389(2)	C(73)-B(1)	1.662(2)
C(74)-C(75)	1.377(2)	C(75)-C(76)	1.378(3)
C(76)-C(77)	1.373(3)	C(77)-C(78)	1.385(2)
O(2B)-C(84C)	1.66(2)	O(2B)-C(87C)	1.402(14)
C(84A)-H(84A)	0.9800	C(84A)-H(84B)	0.9800
C(84A)-H(84C)	0.9800	C(84A)-C(85A)	1.521(8)
C(84B)-H(84D)	0.9800	C(84B)-H(84E)	0.9800
C(84B)-H(84F)	0.9800	C(84B)-C(85B)	1.468(12)
C(84C)-H(84G)	0.9900	C(84C)-H(84H)	0.9900
C(84C)-C(85C)	1.33(2)	C(85A)-H(85A)	0.9900
C(85A)-H(85B)	0.9900	C(85A)-C(86A)	1.558(10)
C(85B)-H(85C)	0.9900	C(85B)-H(85D)	0.9900
C(85B)-C(86B)	1.507(12)	C(85C)-H(85E)	0.9900
C(85C)-H(85F)	0.9900	C(85C)-C(86C)	1.531(18)
C(86A)-H(86A)	0.9900	C(86A)-H(86B)	0.9900
C(86A)-C(87A)	1.487(8)	C(86B)-H(86C)	0.9900
C(86B)-H(86D)	0.9900	C(86B)-C(87B)	1.524(12)
C(86C)-H(86E)	0.9900	C(86C)-H(86F)	0.9900
C(86C)-C(87C)	1.490(19)	C(87A)-H(87A)	0.9900
C(87A)-H(87B)	0.9900	C(87A)-C(88A)	1.537(10)
C(87B)-H(87C)	0.9900	C(87B)-H(87D)	0.9900
C(87B)-C(88B)	1.497(13)	C(87C)-H(87E)	0.9900
C(87C)-H(87F)	0.9900	C(88A)-H(88A)	0.9800
C(88A)-H(88B)	0.9800	C(88A)-H(88C)	0.9800
C(88B)-H(88D)	0.9800	C(88B)-H(88E)	0.9800
C(88B)-H(88F)	0.9800	C(79A)-H(79A)	0.9800
C(79A)-H(79B)	0.9800	C(79A)-H(79C)	0.9800
C(79A)-C(80A)	1.529(8)	C(80A)-H(80A)	0.9900
C(80A)-H(80B)	0.9900	C(80A)-C(81A)	1.529(6)
C(81A)-H(81A)	0.9900	C(81A)-H(81B)	0.9900
C(81A)-C(82A)	1.522(6)	C(82A)-H(82A)	0.9900
C(82A)-H(82B)	0.9900	C(82A)-C(83A)	1.523(7)
C(83A)-H(83A)	0.9800	C(83A)-H(83B)	0.9800
C(83A)-H(83C)	0.9800		

C(1)-Bi(1)-C(57)	92.76(5)	C(60)-O(1)-B(1)	119.10(12)
C(2)-C(1)-Bi(1)	121.37(10)	C(2)-C(1)-C(12)	117.14(13)
C(12)-C(1)-Bi(1)	120.83(10)	C(1)-C(2)-C(3)	126.39(12)
C(1)-C(2)-C(6)	121.65(13)	C(6)-C(2)-C(3)	111.89(12)
C(2)-C(3)-C(4)	102.67(11)	C(2)-C(3)-C(17)	109.79(11)
C(2)-C(3)-C(28)	113.60(11)	C(17)-C(3)-C(4)	115.12(12)
C(17)-C(3)-C(28)	101.13(11)	C(28)-C(3)-C(4)	114.89(11)
C(3)-C(4)-H(4A)	110.0	C(3)-C(4)-H(4B)	110.0
H(4A)-C(4)-H(4B)	108.4	C(5)-C(4)-C(3)	108.52(11)
C(5)-C(4)-H(4A)	110.0	C(5)-C(4)-H(4B)	110.0
C(6)-C(5)-C(4)	102.66(11)	C(6)-C(5)-C(13)	110.58(12)
C(6)-C(5)-C(14)	111.20(12)	C(13)-C(5)-C(4)	110.69(12)
C(13)-C(5)-C(14)	109.36(12)	C(14)-C(5)-C(4)	112.22(12)
C(2)-C(6)-C(5)	112.02(12)	C(7)-C(6)-C(2)	120.49(13)
C(7)-C(6)-C(5)	127.34(13)	C(6)-C(7)-H(7)	120.8
C(6)-C(7)-C(8)	118.49(13)	C(8)-C(7)-H(7)	120.8
C(7)-C(8)-C(9)	127.77(13)	C(12)-C(8)-C(7)	120.35(13)
C(12)-C(8)-C(9)	111.80(13)	C(8)-C(9)-C(10)	102.99(12)
C(8)-C(9)-C(15)	110.41(13)	C(8)-C(9)-C(16)	111.16(13)
C(15)-C(9)-C(10)	110.77(13)	C(15)-C(9)-C(16)	108.68(13)
C(16)-C(9)-C(10)	112.77(13)	C(9)-C(10)-H(10A)	110.0
C(9)-C(10)-H(10B)	110.0	H(10A)-C(10)-H(10B)	108.3
C(11)-C(10)-C(9)	108.67(12)	C(11)-C(10)-H(10A)	110.0
C(11)-C(10)-H(10B)	110.0	C(12)-C(11)-C(10)	102.36(12)
C(12)-C(11)-C(37)	112.14(12)	C(12)-C(11)-C(48)	110.21(12)
C(37)-C(11)-C(10)	116.22(12)	C(37)-C(11)-C(48)	101.15(12)
C(48)-C(11)-C(10)	115.07(12)	C(1)-C(12)-C(11)	126.00(13)
C(8)-C(12)-C(1)	121.80(13)	C(8)-C(12)-C(11)	112.16(12)
C(5)-C(13)-H(13A)	109.5	C(5)-C(13)-H(13B)	109.5
C(5)-C(13)-H(13C)	109.5	H(13A)-C(13)-H(13B)	109.5
H(13A)-C(13)-H(13C)	109.5	H(13B)-C(13)-H(13C)	109.5
C(5)-C(14)-H(14A)	109.5	C(5)-C(14)-H(14B)	109.5
C(5)-C(14)-H(14C)	109.5	H(14A)-C(14)-H(14B)	109.5
H(14A)-C(14)-H(14C)	109.5	H(14B)-C(14)-H(14C)	109.5
C(9)-C(15)-H(15A)	109.5	C(9)-C(15)-H(15B)	109.5
C(9)-C(15)-H(15C)	109.5	H(15A)-C(15)-H(15B)	109.5

H(15A)-C(15)-H(15C)	109.5	H(15B)-C(15)-H(15C)	109.5
C(9)-C(16)-H(16A)	109.5	C(9)-C(16)-H(16B)	109.5
C(9)-C(16)-H(16C)	109.5	H(16A)-C(16)-H(16B)	109.5
H(16A)-C(16)-H(16C)	109.5	H(16B)-C(16)-H(16C)	109.5
C(18)-C(17)-C(3)	127.96(13)	C(18)-C(17)-C(22)	121.04(13)
C(22)-C(17)-C(3)	110.99(12)	C(17)-C(18)-H(18)	119.9
C(17)-C(18)-C(19)	120.27(13)	C(19)-C(18)-H(18)	119.9
C(18)-C(19)-C(20)	117.99(14)	C(18)-C(19)-C(29)	122.68(14)
C(20)-C(19)-C(29)	119.25(14)	C(19)-C(20)-H(20)	118.7
C(21)-C(20)-C(19)	122.69(14)	C(21)-C(20)-H(20)	118.7
C(20)-C(21)-H(21)	120.7	C(20)-C(21)-C(22)	118.60(14)
C(22)-C(21)-H(21)	120.7	C(17)-C(22)-C(21)	119.41(13)
C(17)-C(22)-C(23)	108.49(12)	C(21)-C(22)-C(23)	132.07(13)
C(24)-C(23)-C(22)	130.94(13)	C(24)-C(23)-C(28)	120.30(13)
C(28)-C(23)-C(22)	108.71(12)	C(23)-C(24)-H(24)	120.7
C(25)-C(24)-C(23)	118.61(14)	C(25)-C(24)-H(24)	120.7
C(24)-C(25)-H(25)	118.9	C(24)-C(25)-C(26)	122.15(14)
C(26)-C(25)-H(25)	118.9	C(25)-C(26)-C(33)	118.91(14)
C(27)-C(26)-C(25)	118.40(14)	C(27)-C(26)-C(33)	122.69(14)
C(26)-C(27)-H(27)	119.9	C(28)-C(27)-C(26)	120.13(14)
C(28)-C(27)-H(27)	119.9	C(23)-C(28)-C(3)	110.59(12)
C(27)-C(28)-C(3)	129.00(13)	C(27)-C(28)-C(23)	120.40(13)
C(19)-C(29)-C(30)	110.23(14)	C(19)-C(29)-C(31)	107.67(13)
C(19)-C(29)-C(32)	112.28(13)	C(30)-C(29)-C(31)	109.94(14)
C(32)-C(29)-C(30)	108.43(15)	C(32)-C(29)-C(31)	108.27(15)
C(29)-C(30)-H(30A)	109.5	C(29)-C(30)-H(30B)	109.5
C(29)-C(30)-H(30C)	109.5	H(30A)-C(30)-H(30B)	109.5
H(30A)-C(30)-H(30C)	109.5	H(30B)-C(30)-H(30C)	109.5
C(29)-C(31)-H(31A)	109.5	C(29)-C(31)-H(31B)	109.5
C(29)-C(31)-H(31C)	109.5	H(31A)-C(31)-H(31B)	109.5
H(31A)-C(31)-H(31C)	109.5	H(31B)-C(31)-H(31C)	109.5
C(29)-C(32)-H(32A)	109.5	C(29)-C(32)-H(32B)	109.5
C(29)-C(32)-H(32C)	109.5	H(32A)-C(32)-H(32B)	109.5
H(32A)-C(32)-H(32C)	109.5	H(32B)-C(32)-H(32C)	109.5
C(26)-C(33)-C(36)	109.57(14)	C(34)-C(33)-C(26)	109.33(14)
C(34)-C(33)-C(36)	108.34(15)	C(35)-C(33)-C(26)	111.96(14)

C(35)-C(33)-C(34)	109.14(18)	C(35)-C(33)-C(36)	108.43(18)
C(33)-C(34)-H(34A)	109.5	C(33)-C(34)-H(34B)	109.5
C(33)-C(34)-H(34C)	109.5	H(34A)-C(34)-H(34B)	109.5
H(34A)-C(34)-H(34C)	109.5	H(34B)-C(34)-H(34C)	109.5
C(33)-C(35)-H(35A)	109.5	C(33)-C(35)-H(35B)	109.5
C(33)-C(35)-H(35C)	109.5	H(35A)-C(35)-H(35B)	109.5
H(35A)-C(35)-H(35C)	109.5	H(35B)-C(35)-H(35C)	109.5
C(33)-C(36)-H(36A)	109.5	C(33)-C(36)-H(36B)	109.5
C(33)-C(36)-H(36C)	109.5	H(36A)-C(36)-H(36B)	109.5
H(36A)-C(36)-H(36C)	109.5	H(36B)-C(36)-H(36C)	109.5
C(38)-C(37)-C(11)	129.06(14)	C(38)-C(37)-C(42)	120.40(15)
C(42)-C(37)-C(11)	110.43(13)	C(37)-C(38)-H(38)	119.7
C(37)-C(38)-C(39)	120.58(16)	C(39)-C(38)-H(38)	119.7
C(38)-C(39)-C(40)	117.76(15)	C(38)-C(39)-C(49)	122.26(17)
C(40)-C(39)-C(49)	119.74(16)	C(39)-C(40)-H(40)	118.6
C(41)-C(40)-C(39)	122.77(16)	C(41)-C(40)-H(40)	118.6
C(40)-C(41)-H(41)	120.6	C(40)-C(41)-C(42)	118.88(16)
C(42)-C(41)-H(41)	120.6	C(37)-C(42)-C(43)	108.61(13)
C(41)-C(42)-C(37)	119.36(15)	C(41)-C(42)-C(43)	131.88(15)
C(44)-C(43)-C(42)	131.71(15)	C(44)-C(43)-C(48)	119.91(15)
C(48)-C(43)-C(42)	108.30(13)	C(43)-C(44)-H(44)	120.7
C(45)-C(44)-C(43)	118.66(15)	C(45)-C(44)-H(44)	120.7
C(44)-C(45)-H(45)	118.9	C(44)-C(45)-C(46)	122.17(15)
C(46)-C(45)-H(45)	118.9	C(45)-C(46)-C(53)	121.91(14)
C(47)-C(46)-C(45)	117.92(15)	C(47)-C(46)-C(53)	120.01(15)
C(46)-C(47)-H(47)	119.7	C(48)-C(47)-C(46)	120.53(15)
C(48)-C(47)-H(47)	119.7	C(43)-C(48)-C(11)	111.32(13)
C(47)-C(48)-C(11)	127.84(14)	C(47)-C(48)-C(43)	120.69(14)
C(39)-C(49)-C(50)	106.23(15)	C(39)-C(49)-C(51)	111.70(17)
C(51)-C(49)-C(50)	108.2(2)	C(52)-C(49)-C(39)	112.32(16)
C(52)-C(49)-C(50)	110.2(2)	C(52)-C(49)-C(51)	108.08(16)
C(49)-C(50)-H(50A)	109.5	C(49)-C(50)-H(50B)	109.5
C(49)-C(50)-H(50C)	109.5	H(50A)-C(50)-H(50B)	109.5
H(50A)-C(50)-H(50C)	109.5	H(50B)-C(50)-H(50C)	109.5
C(49)-C(51)-H(51A)	109.5	C(49)-C(51)-H(51B)	109.5
C(49)-C(51)-H(51C)	109.5	H(51A)-C(51)-H(51B)	109.5

H(51A)-C(51)-H(51C)	109.5	H(51B)-C(51)-H(51C)	109.5
C(49)-C(52)-H(52A)	109.5	C(49)-C(52)-H(52B)	109.5
C(49)-C(52)-H(52C)	109.5	H(52A)-C(52)-H(52B)	109.5
H(52A)-C(52)-H(52C)	109.5	H(52B)-C(52)-H(52C)	109.5
C(54)-C(53)-C(46)	112.31(14)	C(54)-C(53)-C(55)	106.16(16)
C(55)-C(53)-C(46)	111.29(15)	C(56)-C(53)-C(46)	107.57(15)
C(56)-C(53)-C(54)	109.24(18)	C(56)-C(53)-C(55)	110.3(2)
C(53)-C(54)-H(54A)	109.5	C(53)-C(54)-H(54B)	109.5
C(53)-C(54)-H(54C)	109.5	H(54A)-C(54)-H(54B)	109.5
H(54A)-C(54)-H(54C)	109.5	H(54B)-C(54)-H(54C)	109.5
C(53)-C(55)-H(55A)	109.5	C(53)-C(55)-H(55B)	109.5
C(53)-C(55)-H(55C)	109.5	H(55A)-C(55)-H(55B)	109.5
H(55A)-C(55)-H(55C)	109.5	H(55B)-C(55)-H(55C)	109.5
C(53)-C(56)-H(56A)	109.5	C(53)-C(56)-H(56B)	109.5
C(53)-C(56)-H(56C)	109.5	H(56A)-C(56)-H(56B)	109.5
H(56A)-C(56)-H(56C)	109.5	H(56B)-C(56)-H(56C)	109.5
Bi(1)-C(57)-H(57A)	108.5	Bi(1)-C(57)-H(57B)	108.5
H(57A)-C(57)-H(57B)	107.5	C(58)-C(57)-Bi(1)	115.21(10)
C(58)-C(57)-H(57A)	108.5	C(58)-C(57)-H(57B)	108.5
C(57)-C(58)-H(58A)	109.8	C(57)-C(58)-H(58B)	109.8
C(57)-C(58)-C(59)	109.49(12)	H(58A)-C(58)-H(58B)	108.2
C(59)-C(58)-H(58A)	109.8	C(59)-C(58)-H(58B)	109.8
C(58)-C(59)-H(59A)	108.7	C(58)-C(59)-H(59B)	108.7
H(59A)-C(59)-H(59B)	107.6	C(60)-C(59)-C(58)	114.29(13)
C(60)-C(59)-H(59A)	108.7	C(60)-C(59)-H(59B)	108.7
O(1)-C(60)-C(59)	108.90(12)	O(1)-C(60)-H(60A)	109.9
O(1)-C(60)-H(60B)	109.9	C(59)-C(60)-H(60A)	109.9
C(59)-C(60)-H(60B)	109.9	H(60A)-C(60)-H(60B)	108.3
C(62)-C(61)-B(1)	118.73(13)	C(66)-C(61)-C(62)	113.30(14)
C(66)-C(61)-B(1)	127.81(14)	F(1)-C(62)-C(61)	119.94(14)
F(1)-C(62)-C(63)	115.42(14)	C(63)-C(62)-C(61)	124.62(15)
F(2)-C(63)-C(62)	120.60(16)	F(2)-C(63)-C(64)	120.06(15)
C(64)-C(63)-C(62)	119.33(16)	F(3)-C(64)-C(63)	120.48(17)
F(3)-C(64)-C(65)	120.72(17)	C(65)-C(64)-C(63)	118.79(15)
F(4)-C(65)-C(64)	119.60(16)	F(4)-C(65)-C(66)	120.44(17)
C(64)-C(65)-C(66)	119.95(16)	F(5)-C(66)-C(61)	121.17(14)

F(5)-C(66)-C(65)	114.83(15)	C(61)-C(66)-C(65)	123.99(16)
C(68)-C(67)-C(72)	113.29(14)	C(68)-C(67)-B(1)	127.18(14)
C(72)-C(67)-B(1)	119.21(13)	F(6)-C(68)-C(67)	121.75(14)
F(6)-C(68)-C(69)	114.43(14)	C(67)-C(68)-C(69)	123.83(15)
F(7)-C(69)-C(68)	120.79(16)	F(7)-C(69)-C(70)	119.43(16)
C(70)-C(69)-C(68)	119.79(16)	F(8)-C(70)-C(69)	120.97(17)
F(8)-C(70)-C(71)	120.25(17)	C(69)-C(70)-C(71)	118.78(15)
F(9)-C(71)-C(70)	119.92(15)	F(9)-C(71)-C(72)	120.67(16)
C(72)-C(71)-C(70)	119.39(15)	F(10)-C(72)-C(67)	119.24(14)
F(10)-C(72)-C(71)	115.95(14)	C(71)-C(72)-C(67)	124.81(15)
C(74)-C(73)-B(1)	117.96(13)	C(78)-C(73)-C(74)	113.15(14)
C(78)-C(73)-B(1)	128.41(14)	F(11)-C(74)-C(73)	119.62(14)
F(11)-C(74)-C(75)	115.64(15)	C(75)-C(74)-C(73)	124.74(15)
F(12)-C(75)-C(74)	120.60(16)	F(12)-C(75)-C(76)	120.07(16)
C(74)-C(75)-C(76)	119.33(16)	F(13)-C(76)-C(75)	120.48(17)
F(13)-C(76)-C(77)	120.66(17)	C(77)-C(76)-C(75)	118.86(16)
F(14)-C(77)-C(76)	119.61(16)	F(14)-C(77)-C(78)	120.58(16)
C(76)-C(77)-C(78)	119.81(16)	F(15)-C(78)-C(73)	121.10(14)
F(15)-C(78)-C(77)	114.79(14)	C(77)-C(78)-C(73)	124.11(15)
O(1)-B(1)-C(61)	106.43(12)	O(1)-B(1)-C(67)	114.01(13)
O(1)-B(1)-C(73)	108.20(13)	C(61)-B(1)-C(67)	102.95(12)
C(73)-B(1)-C(61)	112.31(12)	C(73)-B(1)-C(67)	112.77(12)
C(87C)-O(2B)-C(84C)	101.7(10)	H(84A)-C(84A)-H(84B)	109.5
H(84A)-C(84A)-H(84C)	109.5	H(84B)-C(84A)-H(84C)	109.5
C(85A)-C(84A)-H(84A)	109.5	C(85A)-C(84A)-H(84B)	109.5
C(85A)-C(84A)-H(84C)	109.5	H(84D)-C(84B)-H(84E)	109.5
H(84D)-C(84B)-H(84F)	109.5	H(84E)-C(84B)-H(84F)	109.5
C(85B)-C(84B)-H(84D)	109.5	C(85B)-C(84B)-H(84E)	109.5
C(85B)-C(84B)-H(84F)	109.5	O(2B)-C(84C)-H(84G)	111.9
O(2B)-C(84C)-H(84H)	111.9	H(84G)-C(84C)-H(84H)	109.6
C(85C)-C(84C)-O(2B)	99.2(14)	C(85C)-C(84C)-H(84G)	111.9
C(85C)-C(84C)-H(84H)	111.9	C(84A)-C(85A)-H(85A)	107.9
C(84A)-C(85A)-H(85B)	107.9	C(84A)-C(85A)-C(86A)	117.8(6)
H(85A)-C(85A)-H(85B)	107.2	C(86A)-C(85A)-H(85A)	107.9
C(86A)-C(85A)-H(85B)	107.9	C(84B)-C(85B)-H(85C)	109.3
C(84B)-C(85B)-H(85D)	109.3	C(84B)-C(85B)-C(86B)	111.5(12)

H(85C)-C(85B)-H(85D)	108.0
C(86B)-C(85B)-H(85D)	109.3
C(84C)-C(85C)-H(85F)	111.6
H(85E)-C(85C)-H(85F)	109.4
C(86C)-C(85C)-H(85F)	111.6
C(85A)-C(86A)-H(86B)	109.0
C(87A)-C(86A)-C(85A)	113.0(5)
C(87A)-C(86A)-H(86B)	109.0
C(85B)-C(86B)-H(86D)	110.9
H(86C)-C(86B)-H(86D)	108.9
C(87B)-C(86B)-H(86D)	110.9
C(85C)-C(86C)-H(86F)	111.9
C(87C)-C(86C)-C(85C)	99.2(11)
C(87C)-C(86C)-H(86F)	111.9
C(86A)-C(87A)-H(87B)	108.9
H(87A)-C(87A)-H(87B)	107.7
C(88A)-C(87A)-H(87B)	108.9
C(86B)-C(87B)-H(87D)	109.7
C(88B)-C(87B)-C(86B)	109.8(16)
C(88B)-C(87B)-H(87D)	109.7
O(2B)-C(87C)-H(87E)	110.1
C(86C)-C(87C)-H(87E)	110.1
H(87E)-C(87C)-H(87F)	108.4
C(87A)-C(88A)-H(88B)	109.5
H(88A)-C(88A)-H(88B)	109.5
H(88B)-C(88A)-H(88C)	109.5
C(87B)-C(88B)-H(88E)	109.5
H(88D)-C(88B)-H(88E)	109.5
H(88E)-C(88B)-H(88F)	109.5
H(79A)-C(79A)-H(79C)	109.5
C(80A)-C(79A)-H(79A)	109.5
C(80A)-C(79A)-H(79C)	109.5
C(79A)-C(80A)-H(80B)	109.7
C(81A)-C(80A)-C(79A)	110.0(5)
C(81A)-C(80A)-H(80B)	109.7
C(80A)-C(81A)-H(81B)	109.4
C(86B)-C(85B)-H(85C)	109.3
C(84C)-C(85C)-H(85E)	111.6
C(84C)-C(85C)-C(86C)	100.7(13)
C(86C)-C(85C)-H(85E)	111.6
C(85A)-C(86A)-H(86A)	109.0
H(86A)-C(86A)-H(86B)	107.8
C(87A)-C(86A)-H(86A)	109.0
C(85B)-C(86B)-H(86C)	110.9
C(85B)-C(86B)-C(87B)	104.5(12)
C(87B)-C(86B)-H(86C)	110.9
C(85C)-C(86C)-H(86E)	111.9
H(86E)-C(86C)-H(86F)	109.6
C(87C)-C(86C)-H(86E)	111.9
C(86A)-C(87A)-H(87A)	108.9
C(86A)-C(87A)-C(88A)	113.3(7)
C(88A)-C(87A)-H(87A)	108.9
C(86B)-C(87B)-H(87C)	109.7
H(87C)-C(87B)-H(87D)	108.2
C(88B)-C(87B)-H(87C)	109.7
O(2B)-C(87C)-C(86C)	107.9(10)
O(2B)-C(87C)-H(87F)	110.1
C(86C)-C(87C)-H(87F)	110.1
C(87A)-C(88A)-H(88A)	109.5
C(87A)-C(88A)-H(88C)	109.5
H(88A)-C(88A)-H(88C)	109.5
C(87B)-C(88B)-H(88D)	109.5
C(87B)-C(88B)-H(88F)	109.5
H(88D)-C(88B)-H(88F)	109.5
H(79A)-C(79A)-H(79B)	109.5
H(79B)-C(79A)-H(79C)	109.5
C(80A)-C(79A)-H(79B)	109.5
C(79A)-C(80A)-H(80A)	109.7
H(80A)-C(80A)-H(80B)	108.2
C(81A)-C(80A)-H(80A)	109.7
C(80A)-C(81A)-H(81A)	109.4
H(81A)-C(81A)-H(81B)	108.0

C(82A)-C(81A)-C(80A)	111.4(4)	C(82A)-C(81A)-H(81A)	109.4
C(82A)-C(81A)-H(81B)	109.4	C(81A)-C(82A)-H(82A)	109.0
C(81A)-C(82A)-H(82B)	109.0	C(81A)-C(82A)-C(83A)	113.1(4)
H(82A)-C(82A)-H(82B)	107.8	C(83A)-C(82A)-H(82A)	109.0
C(83A)-C(82A)-H(82B)	109.0	C(82A)-C(83A)-H(83A)	109.5
C(82A)-C(83A)-H(83B)	109.5	C(82A)-C(83A)-H(83C)	109.5
H(83A)-C(83A)-H(83B)	109.5	H(83A)-C(83A)-H(83C)	109.5
H(83B)-C(83A)-H(83C)	109.5		

7. References

[1] Pang, Y.; Nöthling, N.; Leutzsch, M.; Kang, L.; Bill, E.; van Gastel, M.; Reijerse, E. J.; Goddard, R.; Wagner, L.; SantaLucia, D.; DeBeer, S.; Neese, F.; Cornella, J. Synthesis and isolation of a triplet bismuthinidene with a quenched magnetic response. *Science* **2023**, 380, 1043–1048.

[2] Spinnato, D.; Nöthling, N.; Leutzsch, M.; van Gastel, M.; Wagner, L.; Neese, F.; Cornella, J. A trimetallic bismuth(I)-based allyl cation. *Nat. Chem.* **2025**, 17, 265–270.

[3] Kratzert, D.; Krossing, I. Recent improvements in DSR. *J. Appl. Cryst.* **2018**, 51, 928–934.

[4] Dolomanov, O.V.; Bourhis, L.J.; Gildea, R.J; Howard, J.A.K.; Puschmann, H. OLEX2: A Complete Structure Solution, Refinement and Analysis Program. *J. Appl. Cryst.* **2019**, 42, 339–341.

[5] Kratzert, D.; Holstein, J.J.; Krossing, I. DSR: enhanced modelling and refinement of disordered structures with SHELXL. *J. Appl. Cryst.* **2015**, 48, 933–938.
